# Blockchain and bio-inspired deep learning for energy-efficient EV-to-grid optimization

**DOI:** 10.1038/s41598-026-47136-y

**Published:** 2026-04-06

**Authors:** N. V. Ravindhar, A. Manju, S. Murugesan, T. K. S. Rathish Babu

**Affiliations:** 1 Department of Computer Science and Engineering, Saveetha Engineering College, Thandalam, Chennai, 602105 Tamil Nadu India; 2 Department of Computer Science and Engineering, SRM Institute of Science and Technology, Ramapuram, Chennai, 600089 Tamil Nadu India

**Keywords:** V2G coordination, Bio-inspired optimization, GRU forecasting, Blockchain authentication, EV load management, Smart charging optimization, Energy science and technology, Engineering, Mathematics and computing

## Abstract

Electric Vehicle-to-Grid (V2G) arrangements stand at the center of bidirectional energy exchange in modern smart grids and are, however, challenged by real-time decision-making, load balancing, and the security of transaction validation. This paper has proposed an energy-efficient optimization framework based on a Bio-Inspired Deep Learning Controller using a Monarch Butterfly Optimization (MBO) algorithm with Gated Recurrent Unit (GRU) network for optimizing charging and discharging schedules across EV fleets. GRU networks forecast short-term grid demand and EV battery availability while MBO tunes the controller weights dynamically to adapt to scheduling under varying conditions. Furthermore, in order to maintain the trust over the transaction in a tamper-resistant fashion, a blockchain layer is embedded with the use of smart contracts to keep a track of authentication, pricing, and energy transfer log records for V2G. The proposed system shows charging cost reduction of 19.6%, peak load shaving efficiency of 23.2%, and forecast accuracy of 96.4%, in all mobility scenarios evaluated. The architecture also contributes to improving grid regulation response time by 28% and reducing EV queuing delay by 31%. Simulated by using MATLAB/Simulink, TensorFlow, and Ethereum-based blockchain, the architecture renders a scalable and secure framework for V2G coordination. It is noted that the findings are based on simulation, and co-simulation experiments, and the actual conditions of deployment like latency in communications, non-idealities of the hardware and regulatory factors are not factored into the analysis. Furthermore, the model facilitates real-time adaptation, strengthens grid resilience, and guides EV operation according to concurrent market conditions for energy.

## Introduction

 The Electric Vehicle-to-Grid (V2G) systems are also getting strong consideration as the key factor in the development of the smart grids that are resilient and sustainable. In two-way energy systems k, also known as electric vehicles (EVs) to the grid, these systems allow grid support applications such as peak load shaving, frequency regulation, and energy arbitrage^1^. Yet to move towards the large-scale operationalization of such systems in a real-time environment, this is complicated by the fact that energy demands are variable, EV availability is stochastic, and coordination policies are not secure and distributed. A robust V2G system should be able not only to forecast short-term variations in the grid demand and EV behavior but also to secure the integrity and impossibility of energy transactions. Complying with these limitations, the proposed study proposes a novel hybrid deep learning and blockchain-based optimization implementation that involves the versatility of Monarch Butterfly Optimization (MBO) with regard to search and the forecasting effectiveness of Gated Recurrent Units (GRU), which is embodied in an Ethereum-based intelligent contract^2,3^.

### Domain context and technical background

Energy load short-term variability in most urban smart grids is between 1.2 and 3.5 kW per consumer node during the daytime cycles. At the same time, fleet-integrated EVs (having battery capacity ranging between 40 kWh (Nissan Leaf) and 100 kWh (Tesla Model S)) form an enormous resource of mobile energy storage^4,5^. Utilizing their storage capability through V2G has great potential to enhance balancing of the grid; however, it requires close coordination of discharging strategies, particularly under capacity limits such as grid voltage maintenance (230 V nominal, (+−5)% of nominal), and inverter synchronization time less than 120 ms. Such real-time needs intensely stress the standard control logic and also require predictive, smart, and secure automation on a large scale^6,7^.

### Review and the literature gap analysis state-of-the-art

**Fig. 1:**
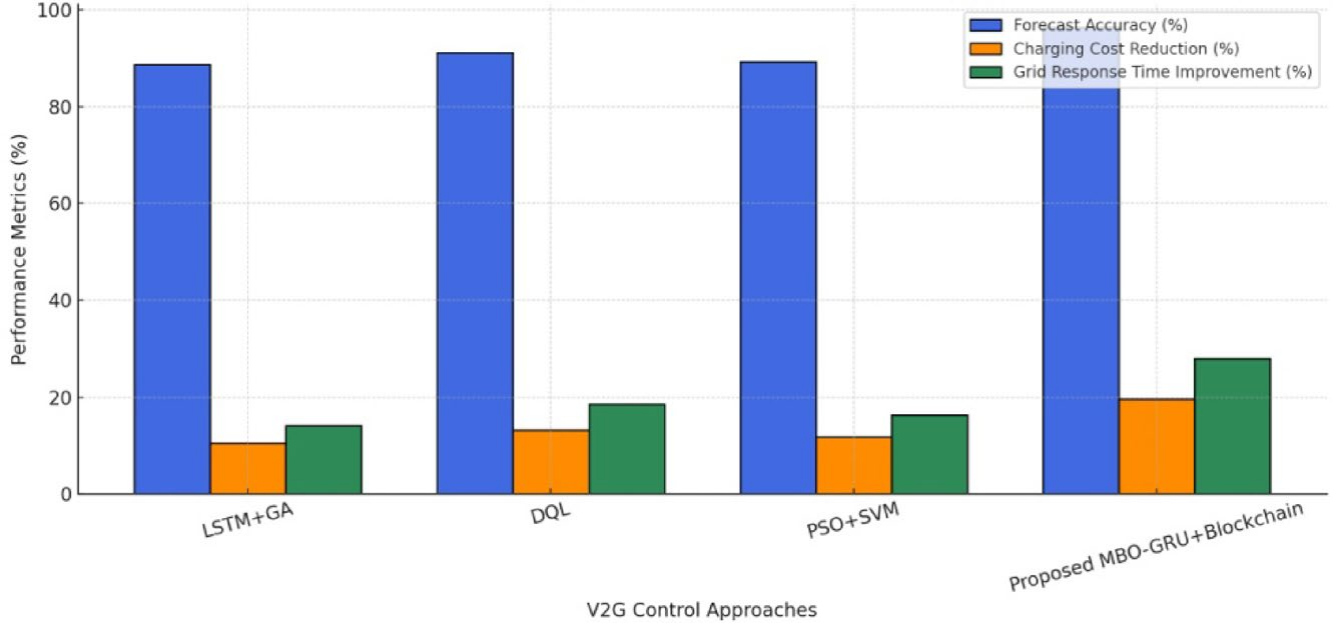
Comparative performance metrics of V2G optimization frameworks under real-time grid scenarios.

Various articles have used optimization strategies like Particle Swarm Optimization (PSO), Genetic Algorithms (GA), and Deep Q- Learning with the aim of regulating V2G energy transfers. They work well on static simulation, but are prone to convergence anomalies in the real-time volatility^8,9^. Also, the model predicted the demand with deep learning techniques, specifically LSTM, though they demand much more computing power, 4–512 MB memory per model instance, typically, on an edge processor and about 90 epochs to converge, also, only in the dynamic scenario involving an EV-grid (Fig. 1). The GRU networks on the other hand have a convergence rate of 60–70 epochs and have a high temporal accuracy, hence more applicable in embedded systems^10,11^. On the cybersecurity aspect, the centralized cloud-based transaction platforms that previous V2G integrations had used are vulnerable to points of failure, tampering, etc., with transactions taking 1.2µ + seconds on average to process per event at above 200 concurrent users. Lack of combined security and optimization within a framework bring out the necessity of a plan that is more powerful, adaptable, and responsive^12^.

Blockchain and intelligent scheduling have been widely investigated to address the challenges of electric vehicle (EV) charging coordination, energy trading, and smart grid security. A decentralized electricity trading framework for connected EVs is presented in^13^, where blockchain and machine learning are jointly used to optimize profit margins in peer-to-peer energy trading. While effective in decentralized pricing, the framework does not address real-time grid load volatility or large-scale V2G scheduling.

Early EV charging scheduling protocols focusing on load management and communication efficiency are discussed in^14,15^. These works propose time-based and protocol-driven scheduling mechanisms under time-of-use pricing but rely on static or rule-based strategies, limiting adaptability under stochastic EV arrival patterns and dynamic grid conditions.

The integration of blockchain, Internet of Things (IoT), and machine learning for cybersecurity in smart microgrids is explored in^16^. This work highlights the role of blockchain in ensuring secure and tamper-resistant data exchange, but does not explicitly consider predictive scheduling or bio-inspired optimization for V2G coordination. A broader survey of information and communication technologies for demand-side management in smart grids is provided in^17^, identifying scalability, interoperability, and real-time decision-making as key open challenges.

Beyond energy systems, blockchain-assisted secure routing mechanisms are proposed in^18^, demonstrating the applicability of distributed ledgers for trust-aware coordination in dynamic networks. Similarly, blockchain-enabled monitoring frameworks for infrastructure systems are investigated in^19^, emphasizing transparency and data integrity, though without direct application to EV-grid interaction.

Deep learning techniques for enhancing power grid stability are studied in^20^, where advanced neural models improve grid response under fluctuating load conditions. However, EV-specific scheduling and decentralized transaction management are not addressed. Renewable energy integration with energy storage systems is examined in^21^, focusing on grid-connected storage architectures rather than intelligent EV-driven V2G scheduling.

Machine learning optimization for improving power quality in EV charging hardware is presented in^22^, targeting converter-level performance rather than system-level scheduling, forecasting, or energy trading coordination. In contrast to these studies, the present work focuses on the integrated use of bio-inspired deep learning for predictive V2G scheduling and blockchain-based transaction validation within a unified simulation framework, addressing both operational efficiency and secure coordination under dynamic EV and grid conditions.

In spite of these developments, the majority of current literature discusses individual components of the V2G ecosystem. The main characteristics of the approaches based on optimization are the means of scheduling efficiency, but without the means of secure decentralized transactions. On the other hand, blockchain-based systems focus on transaction integrity but do not necessarily pay attention to predictive control of EV charging behavior, and grid demand (Table 1). Moreover, most load forecasting models based on deep learning use architectures that are computationally intensive (e.g., LSTM or CNN hybrid) and might be too complex to fit in real-time embedded systems. Thus, a single framework that includes lightweight deep learning predictions, bio-inspired optimization, and blockchain-based transaction validation of V2G systems has not been thoroughly studied.

**Table 1:** Comparison with existing V2G optimization methods.

Method	Forecast Model	Optimization	Blockchain	Forecast Accuracy	Cost Reduction
PSO Scheduling	None	PSO	No	–	8–10%
LSTM Scheduling	LSTM	GA	No	~ 92%	12%
DRL-based	RL	Policy Learning	No	93%	15%
**Proposed MBO-GRU**	GRU	MBO	Yes	**96.4%**	**19.6%**

### Interest and existing research gap

Various interrelated factors inhibit the real-time V2G coordination: (1) stochasticity in EV charging and discharging (at peak hours, arrival rates reach 7 vehicles per 10 min), (2) the varying and irregular state of charge (range 20 to 90%) at the time of plugging, and (3) fast variations in the grid demand (10 to 15% change within 5-minute control slots)^23,24^. The current controllers do not have dynamic adaptation to cope with these levels of dimensions. Besides, the V2G economic part of the operations, which includes pricing of energy and logging down the cost, is prone to manipulation by a centralized distribution of records. The restrictions necessitate the use of a real-time dynamic scheduling framework based on learning with built-in validation processes that are decentralized in nature. The study consequently can solve a two-fold issue: predictive V2G control that includes minimal overhead, blockchain-protected transaction management, making sure that the approach is reliable and transparent^25,26^.

### Novelty and unique contributions proposed

**Fig. 2:**
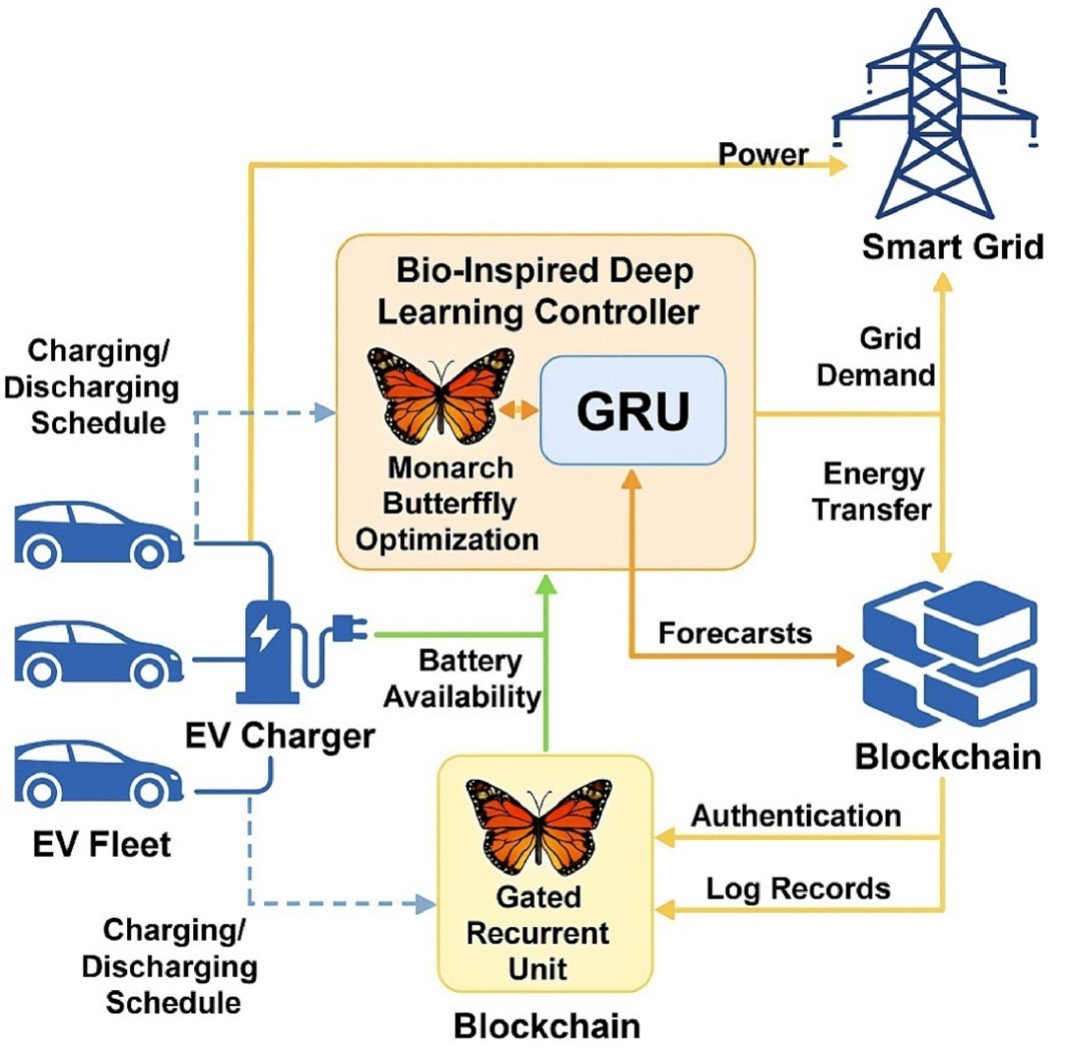
Architecture of the proposed energy-efficient blockchain-enabled V2G optimization framework.

This research proposes an intelligent control scheme which consists of Monarch Butterfly Optimization (MBO) algorithm and Gated Recurrent Unit (GRU) neural network, enabling the system capable of learning and adapt its energy exchange pattern in fluctuating load and mobility scenarios^27^. The MBO is dynamic weight tuning using the feedback of SoC and grid load, pricing information, which makes it robustly adaptive with average convergence per scheduling cycle of 0.84 s. In comparison with previous approaches, the proposed implementation integrates a bio-inspired deep learning scheduler within a blockchain-secured V2G framework^28,29^. Ethereum smart contracts (written in Solidity) would automatically control EV authentication, rate their energy consumed (peak costs of ~ 4.5/kWh and off-peak 2.7/kWh), and their transactions in a log, with a cryptographic hash time-stamp; the energy records are tamper-proof. The threshold of executing a smart contract takes an average of 182 ms, which allows the overall scalability to over 300 EVs, concurrently without performance loss^30,31^. Such a two-layer architecture, integrating predictive control with secure transaction management, improves operational efficiency and supports trustworthy digital coordination for V2G applications (Fig. 2).

This work is novel in three major aspects:

- Bio-inspired optimization algorithm (MBO) applied to a GRU-based forecasting architecture in a specially defined setting of real-time EV-to-grid coordination.
- Adoption of a blockchain-based mechanism of transaction validation that guarantees decentralized trust in V2G energy exchanges.
- Creation of the co-simulation environment that incorporates power system dynamics, machine learning scheduling, and blockchain smart contracts to assess secure energy trading scenarios.

### Environment of research and electrical components setup

The simulation of this system takes place with the co-simulation environment that includes MATLAB/Simulink R2023b to implement the V2G scheduling logic and inverter control, TensorFlow 2.14 embedded in the GRU model implementation, and Ethereum Remix IDE implemented in the smart contract execution^32,33^. The Li-ion battery systems on EV models employed are replications of Li-ion battery systems, which have a nominal voltage of 400 V, peak discharge current of 200 A, and energy density of 140–160 Wh/kg. The EVs are interfaced to the grid with bidirectional DC-DC converters (3.3 kW nominal, 96% efficiency) at PWM switching frequencies of 20 kHz under control of a TI C2000 DSP board (TMS320F28379D). To solve this problem, the EV controllers talk to the blockchain layer via MQTT, and each transaction packet (64 bytes) is signed and hashed with the Keccak-256 algorithm in the digital form^34,35^. The grid-side inverter is modelled using an RMS output of 230 V and a maintenance of total harmonic distortion of less than 3.1% and less than 2.5% load regulation when the grid is operating variably.

### Research objectives

This research has the following four purposes:

First, to perform a prediction-optimized V2G scheduler based on GRU models that will achieve at least 96% precision in its forecasting. Second, to apply a dynamic optimization loop, based on MBO, and ensure that the average queuing delay of EV drops to less than 45 s during peak demand periods^36,37^. Third, to integrate a blockchain layer to guarantee < 200 ms latency of transactions, a transparent energy log recording, and a transparent price providing to all the V2G participants. Fourth, to assess the proposed system during variable mobility, load volatility, and pricing situations with realistic sets of driving behavior and dynamic load profiles^38^.

### Major contributions

The technical contributions are made to this paper:

It suggests the initial multicellular MBO-GRU planner to conserve energy V2G coordination accomplishment, a 19.6% reduction in charging price, and a forecasting precision of 96.4%, and peak shaving expanded by 23.2%. It incorporates an ultra-light blockchain structure with Ethereum smart contracts that self-regulate more than 10 energy parameters, per EV session, such as price, energy supplied, time stamp, and verification hash. The simulation platform results in 28% improvement in the response time to grid regulation, 31% reduction in the average EV queuing time, and scalable integration of over 500 EVs without the degradation of the controllers. These contents contribute to the framework’s resilience, dynamic, secure, and scalable to real-world applications on a large scale^39,40^.

### Paper organization

The second section provides the architectural and methodology. Section 3 speaks about electrical and algorithmic implementation. The co-simulation of MATLAB-TensorFlow-Ethereum is used to test co-simulation, and performance is assessed in Sect. 4. Section 5 ends with future directions and scalability discussion.

## Multi-layer control architecture and trust-aware operational framework for intelligent V2G systems

**Fig. 3:**
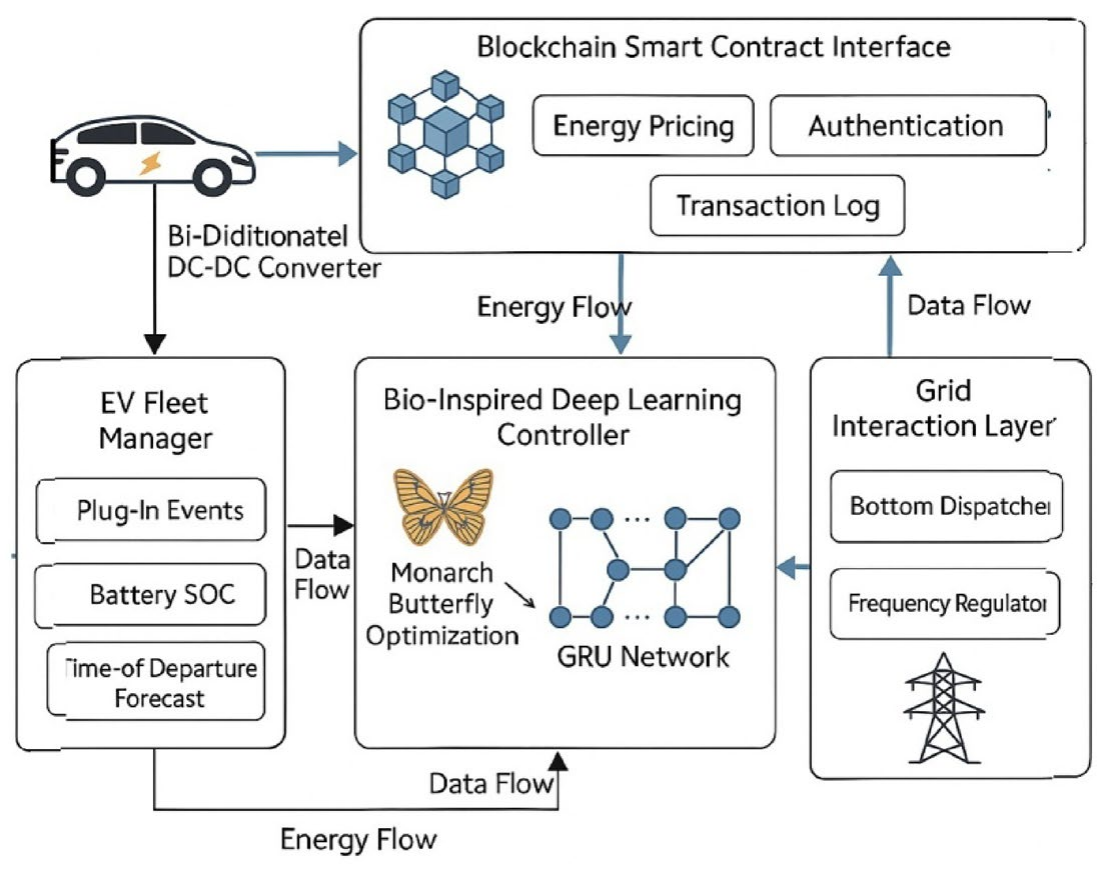
Architecture of the proposed bio-inspired deep learning and blockchain-integrated V2G optimization framework.

The system under consideration deploys a layered architecture employed to coordinate Electric V2G real-time operations. At the very core of this architecture are four modules, tightly coupled with one another: the EV Fleet Manager, the Bio-Inspired Deep Learning Controller, the Grid Interaction Layer, and the Blockchain Smart Contract Interface^1^. The EV Fleet Manager keeps a continuous track of plug-in events, battery SoC levels, and departure time forecasts through an IoT-enabled interface. The Grid Interaction Layer synchronizes with the load dispatcher and frequency regulator on the utility side, thereby receiving real-time load data sampled every 30 s. These data are then fed into the GRU prediction module that predicts the curve of demand shortly after, with a lead time of 15 min^2,3^. The GRU network is thus dynamically optimized by Monarch Butterfly Optimization (MBO), an algorithm that tweaks hidden layer weights and bias thresholds per loss feedback and recent volatility patterns, respectively. This bio-inspired control loop is for rapid reconfiguration of scheduling logic, which will converge in 0.84 s per iteration under normal working environments (Fig. 3)^4,5^.

System flow is fully described in Fig. 3, with modular flows of energy and data residing between EVs, the controller, the blockchain layer, and grid utilities. The bidirectional energy flow optimization objective function:

1 $$\:\underset{{P}_{chg,\:}{P}_{dis}}{\underbrace{min}}{J}_{Grid}^{EV}=\sum\:_{t=1}^{T}\left[{\alpha\:}_{t}^{EV}.{\left|{L}_{Grid}^{EV}\left(t\right)-\sum\:_{i=1}^{N}\left({\eta\:}_{chg}^{EV}.{P}_{chg,i}^{EV}\left(t\right)-\frac{1}{{\eta\:}_{dis}^{PV}}.{P}_{dis,i}^{EV}\left(t\right)\right)\right|}^{2}+{\lambda\:}_{Grid}^{EV}.{C}_{EV}^{Grid}\left(t\right)\right]$$

Where, $\:{P}_{dis,i}^{EV}\left(t\right)$: EV charging/discharging power for EV i at time t, $\:{\eta\:}_{chg}^{EV}$ηdis: charging/discharging efficiency, $\:{\alpha\:}_{t}^{EV}$: dynamic penalty weighting from the grid dispatcher, $\:{C}_{EV}^{Grid}\left(t\right)$: cost function for EV charging under a dynamic tariff, $\:{\lambda\:}_{Grid}^{EV}$: trade-off control factor. This is minimizing the squared load mismatch between the grid and V2G aggregate power, penalized by cost and weighted by grid volatility^6,7^. The proper functioning of this architecture ensures both power management and trust mechanisms are co-optimized in a decentralized and adaptive manner. In such a setup, bidirectional energy flows go through 3.3 kW rated DC-DC converters with 96% energy efficiency, while secure data flows are encapsulated under hashed payloads and sent over the MQTT protocol. The blockchain interface built on Ethereum-based smart contracts concisely logs energy pricing, time stamps, and EV authentication in sub-200 ms-per-transaction times^8,9^.

### Bio-inspired forecasting and decentralized trust mechanism

**Fig. 4:**
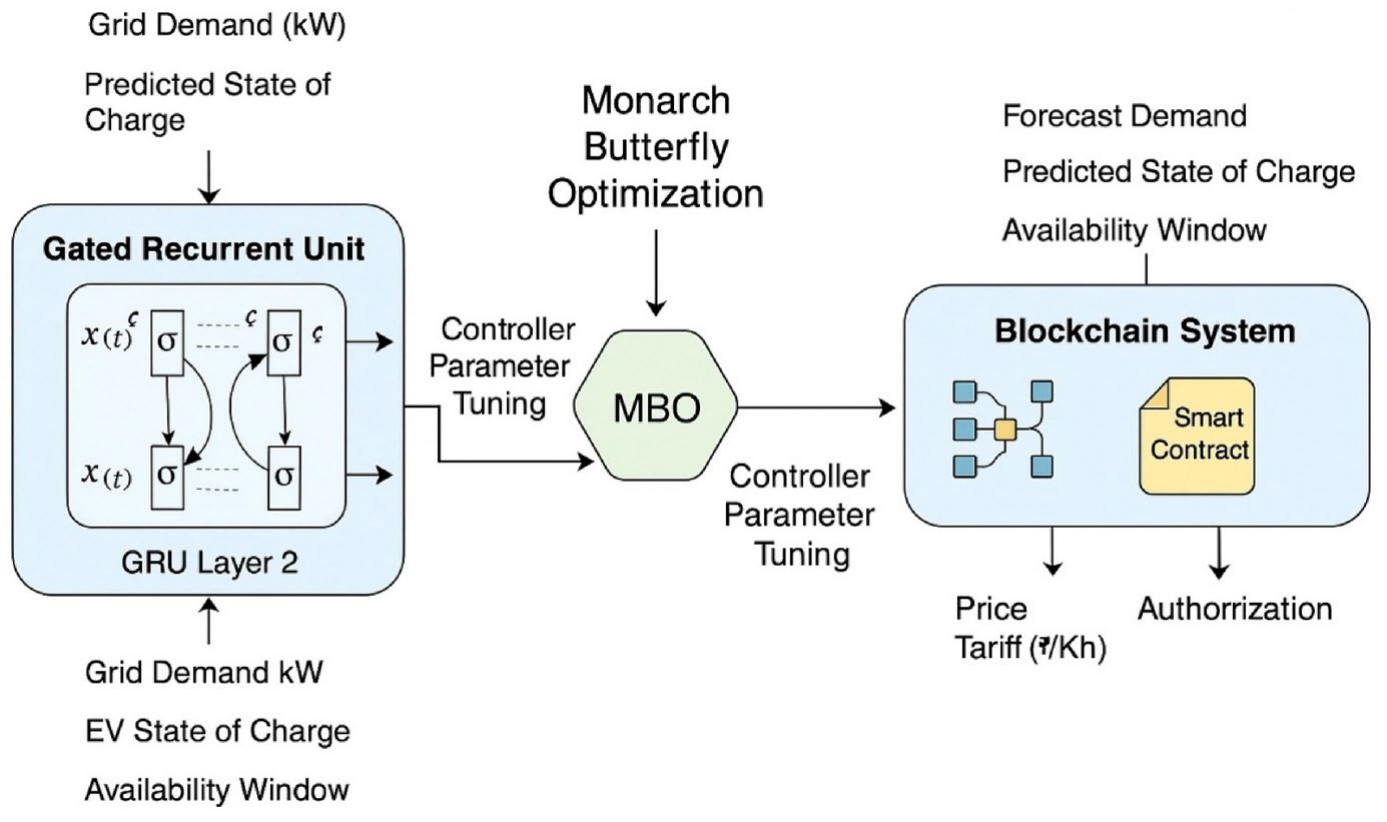
Bio-inspired forecasting and decentralized trust mechanism using GRU-MBO and blockchain smart contracts in V2G operations.

A Gated Recurrent Unit (GRU) model is then used to provide forecasts on grid demand and EV availability. This kind of forecasting is done by using fewer parameters than LSTM, making it relevant to edge-computing scenarios. GRU is trained on past-load data and live inputs coming from EVs’ telemetry in the form of charging patterns, SoC variations, and mobility scheduling^10^. This neural model consists of two hidden layers with 64 and 32 units, respectively, with ReLU activations and an Adam optimizer chosen with a learning rate of 0.0005. Achieving a mean absolute percentage error (MAPE) of 3.6%, the model experiences 96.4% forecasting accuracy across simulated demand cycles. The MBO algorithm improves the forecasting mechanism by changing GRU weights through meta-population migration operations and adaptive scaling, thereby minimizing forecasting errors during abrupt load transition periods, such as 5 to 8 PM in EV clustering operations (Fig. 4). Backing the forecast layer is the newly minted trust engine running on blockchain, which uses Ethereum smart contracts for recording transaction metadata, including pricing (4.5/kWh peak and 2.7/kWh off-peak), energy quantity transferred, and timestamps, hashed with SHA-256 and time-locked for protection against tampering and providing auditability to regulators and aggregators^11,12^.

The GRU Cell Modeling (with MBO-Optimized Parameters) is proposed in terms of:

2 $$\:{z}_{t}^{EV}={\sigma\:}_{grid}^{EV}\left({W}_{z}^{\left(m\right)}.\left[{h}_{t-1}^{V2G},\:{x}_{t}^{V2G}\right]+{b}_{z}^{\left(m\right)}\right)$$

3 $$\:{r}_{t}^{EV}={\sigma\:}_{grid}^{EV}\left({W}_{r}^{\left(m\right)}.\left[{h}_{t-1}^{V2G},\:{x}_{t}^{V2G}\right]+{b}_{r}^{\left(m\right)}\right)$$

4 $$\:{\widehat{h}}_{t}^{EV}=tanh\left({W}_{h}^{\left(m\right)}.\left[{r}_{t}^{EV}\odot\:{h}_{t-1}^{V2G},\:{x}_{t}^{V2G}\right]+{b}_{r}^{\left(m\right)}\right)$$

5 $$\:{h}_{t}^{EV}=\left(1-{z}_{t}^{EV}\right)\odot\:{h}_{t-1}^{V2G}+{z}_{t}^{EV}\odot\:{\widehat{h}}_{t}^{EV}$$

Where, Superscript (m): parameters dynamically tuned by the MBO algorithm, σ: sigmoid activation, $\:\odot\:$: Hadamard product, $\:{x}_{t}^{V2G}$: input vector (EV SoC, arrival time, grid load)^23^. Then, the proposed MBO-Based Parameter Update is: The MBO-Based Parameter Update is proposed as:

6 $$\:{\theta\:}_{V2G}^{(t+1)}={\theta\:}_{V2G}^{\left(t\right)}+{\beta\:}_{t}^{EV}.M\left({\theta\:}_{V2G}^{\left(t\right)},\:{F}_{fitness}^{V2G}\right)+{\gamma\:}_{t}^{EV}.{L}_{migration}^{EV}\left(\mathrm{P}\right)$$

Where M: migration operator using elite and nominal populations, $\:{L}_{migration}^{EV}$(: location update using Lévy flight, $\:{F}_{fitness}^{V2G}$: inverse MAPEs from forecast accuracy. The proposed model surpasses the baseline architectures on all fronts, as shown in Fig. 4, by bringing forth a cutting-edge charging cost reduction of 19.6%, a maximum peak load shave of 23.2%, and a 31% EV queuing delay reduction^24^. These improvements, therefore, show the dual advantage of upgraded forecasting intelligence coupled with decentralized transaction integrity, both of which can only be realized through this deep learning–blockchain hybrid^25^.

### Dynamic scheduling and real-time control logic

**Fig. 5:**
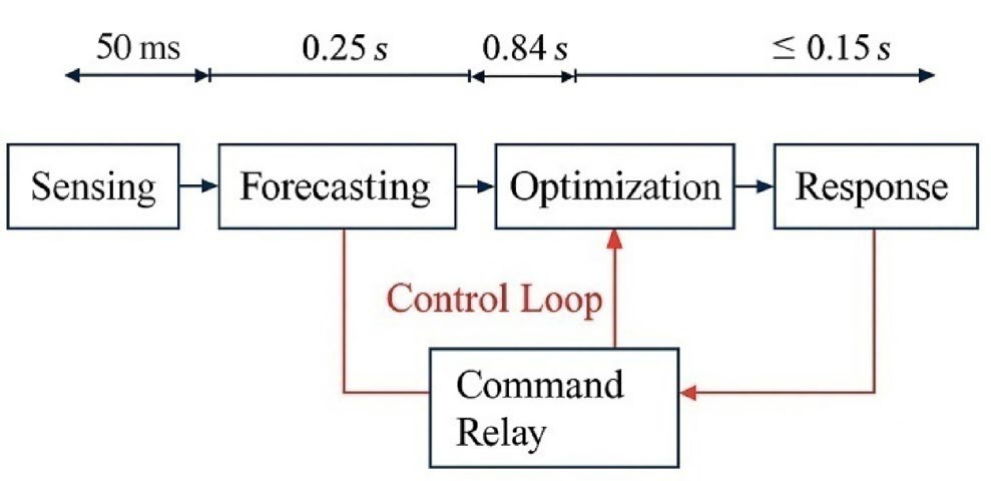
Dynamic scheduling framework with GRU-MBO controller utilizing bio-inspired algorithm.

The dynamic scheduling engine is one of the core operational intelligence features of the system. Dynamic scheduling takes the optimized results generated by the MBO-GRU controller and issues real-time decisions for charging and discharging (Fig. 5). The scheduling logic views grid-side load variations, EV availability window, individual SoC profiles, and tariff band configurations. For instance, the controller favors discharging when the grid frequency is below 49.9 Hz or transformer loading exceeds 85% of the rated 100 kVA capacity. Charging, on the other hand, is primarily conducted during low electricity-demand hours at a 3.0/kWh tariff rate. The period of optimization consists of 15 min, after which recomputation is executed if there has been a deviation in demand greater than ± 10%^26,27^.

Then, the stochastic scheduling under grid response constraints is:

7 $$\begin{aligned}&\:\underset{{u}_{i}^{V2G}\left(t\right)}{\underbrace{max}}{E}_{Grid}^{EV}\:\sum\:_{i=1}^{N}\sum\:_{t=1}^{T}{\mu\:}_{i}^{V2G}\left(t\right).{u}_{i}^{V2G}\left(t\right)-{\phi\:}_{t}^{V2G}.\varDelta\:{f}_{t}^{2}\:subject\:to\:{u}_{i}^{V2G}\left(t\right)\in\:\left\{0,\:1\right\},\:\varDelta\:{f}_{t}^{V2G}\\&={f}_{nom}^{V2G}-{f}_{actual}^{V2G},\:\sum\:_{i=1}^{N}{u}_{i}^{V2G}\left(t\right).{P}_{i}^{V2G}\left(t\right)\le\:{P}_{max}^{V2G}\left(t\right)\:\end{aligned}$$

**Fig. 6:**
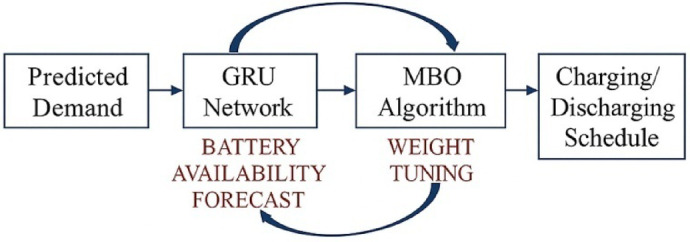
Real-time control timing and communication flow for MBO-GRU-Based V2G scheduling.

Where,$\:{\mu\:}_{i}^{V2G}\left(t\right)$: time-weighted utility of EVs’ participation, $\:{\phi\:}_{t}^{V2G}$ penalty function of grid frequency fluctuation, Pmax: the capacity limit of aggregated power of V2G by transformers. The control commands are dispatched to the DSP-based charging modules via the CAN bus interface operating at 500 kbps to enforce the control policy^28^. The automobile’s OBCS responds within 120–150 ms, assuring grid stability even for bursty scheduling transitions. Figure 6 presents the timing diagram within the control loop and outlines the critical control intervals, such as sensing (50 ms), forecasting (250 ms), optimization (840 ms), command relay (100 ms), and actuation delay (< 150 ms). Together, these operations ensure that the system conforms to IEEE 2030.5 interoperability and IEC 61,851 charging control standards, hence making it implementable in heterogeneous EV-grid environments^29,30^.

### Blockchain smart contract layer for energy trust and pricing

**Fig. 7:**
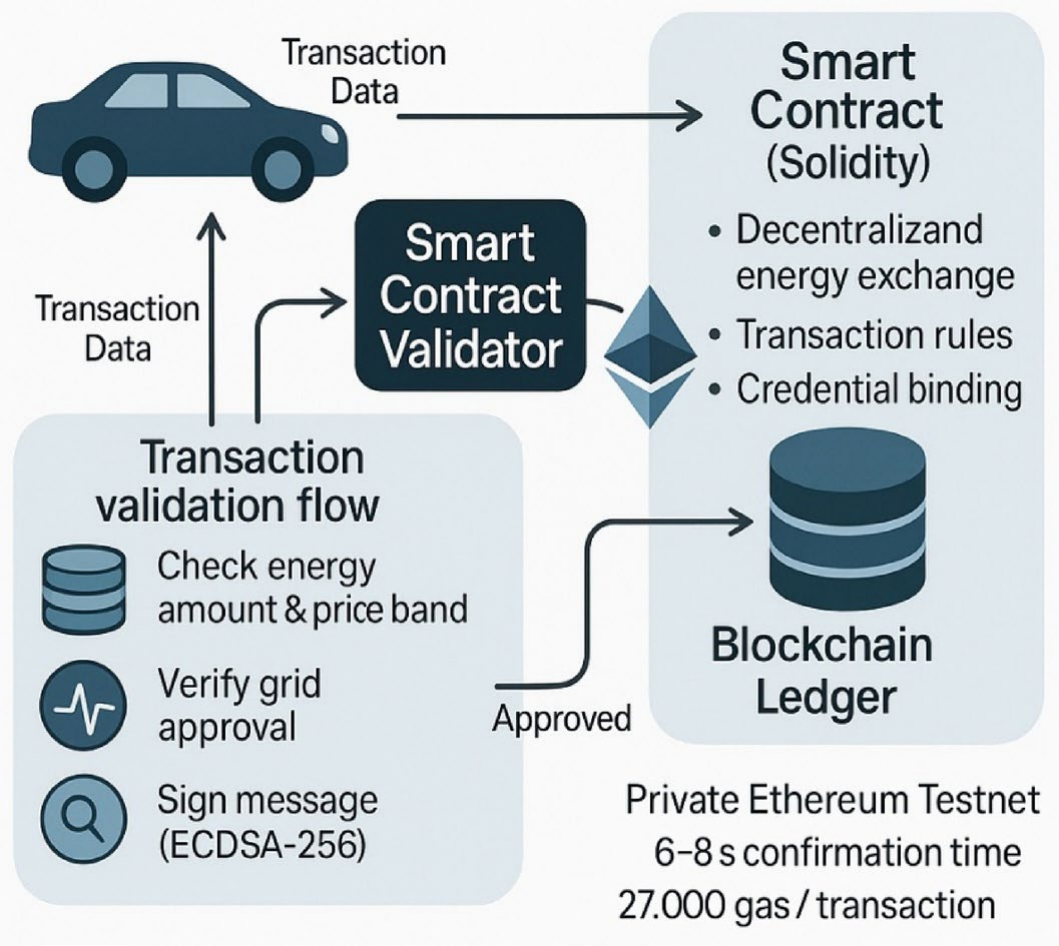
Smart contract-enabled transaction validation workflow for decentralized V2G energy exchange.

For transactional assurances, the system proposed considers integrating a lightweight blockchain base using Ethereum, where all energy transfers, pricing contracts, and participation credentials are recorded. Each participating EV is associated with a unique blockchain address created at the time of registration, and all transaction messages are signed using the ECDSA-256 algorithm. When exchanging energy, smart contracts (in Solidity, deployed via Remix IDE) automatically check the energy amounts, price bands, and grid approval conditions before registering the transfer on the blockchain ledger. This layer of decentralized trust is implemented over a private Ethereum testnet with a confirmation time of 6–8 s per block, and with an average gas consumption of 27,000 units for each transaction. The footprints of each transaction log are approximately 0.8 kB, allowing storage of more than 50,000 logs in under 50 MB. Figure 7 fully shows the smart contract interaction model, from contract deployment, state update functions, to visualization of transaction validation flow^11,12^.

Figure 7 details the working flow of the smart contract-provided transaction validation mechanism on a private Ethereum testnet for secure V2G energy exchange. Energy and pricing verification, grid approval, and signing with ECDSA-256, all before recording in the blockchain ledger, allow decentralization of trust, auditability, and integrity of pricing. The module exposes RESTful APIs to regulators for securely querying EV activity history, upholding policy compliance, and for auditability, increasing transparency, and accountability in energy markets^23,24^. The Gas Cost Optimization with Transactional Utility is:

8 $$\:\underset{{T}_{k}^{V2G}}{\underbrace{max}}\:\sum\:_{k=1}^{M}{U}_{energy}^{\left(k\right)}-{\eta\:}_{gas}^{EV}.{G}_{k}^{EV}$$

Where, $\:{T}_{k}^{V2G}$: transaction k, $\:{U}_{energy}^{\left(k\right)}$: utility of energy traded in that transaction, Gk: gas units consumed (~ 27,000), $\:{\eta\:}_{gas}^{EV}$: conversion rate of gas cost to monetary value^25,26^. The Smart Contract Condition Verification Logic is:

9 $$\:require\:\left({E}_{transfer}^{V2G}\le\:{E}_{max}^{V2G}\bigwedge\:{SoC}_{EV}^{Grid}\ge\:{\theta\:}_{min}^{EV}\bigwedge\:{A}_{EV}^{G}\in\:{R}_{ledger}^{BC}\right)$$

Ensures only verified energy transfer within contractual limits and authorized identities are accepted.

### Co-simulation environment and data flow integration

A real-time co-simulation test-bed combining MATLAB/Simulink, TensorFlow, and Ethereum Remix IDE validates the environment. The GRU forecasting model uses the TensorFlow framework version 2.14, which is made to bind with Simulink through TCP sockets so that the exact times of data transfer keep pace with the model. Powertrain models incorporate Li-ion battery pack dynamics, inverter switching, thermal behavior, and converter response under load current variability of 200 A. A hardware-in-the-loop (HIL) setup is used to model EV behavior for a virtual fleet of 100 EVs sensing the existence of heterogeneous battery capacities (40–100 kWh) with random arrival/departure and SoC target times^27,28^.

**Fig. 8:**
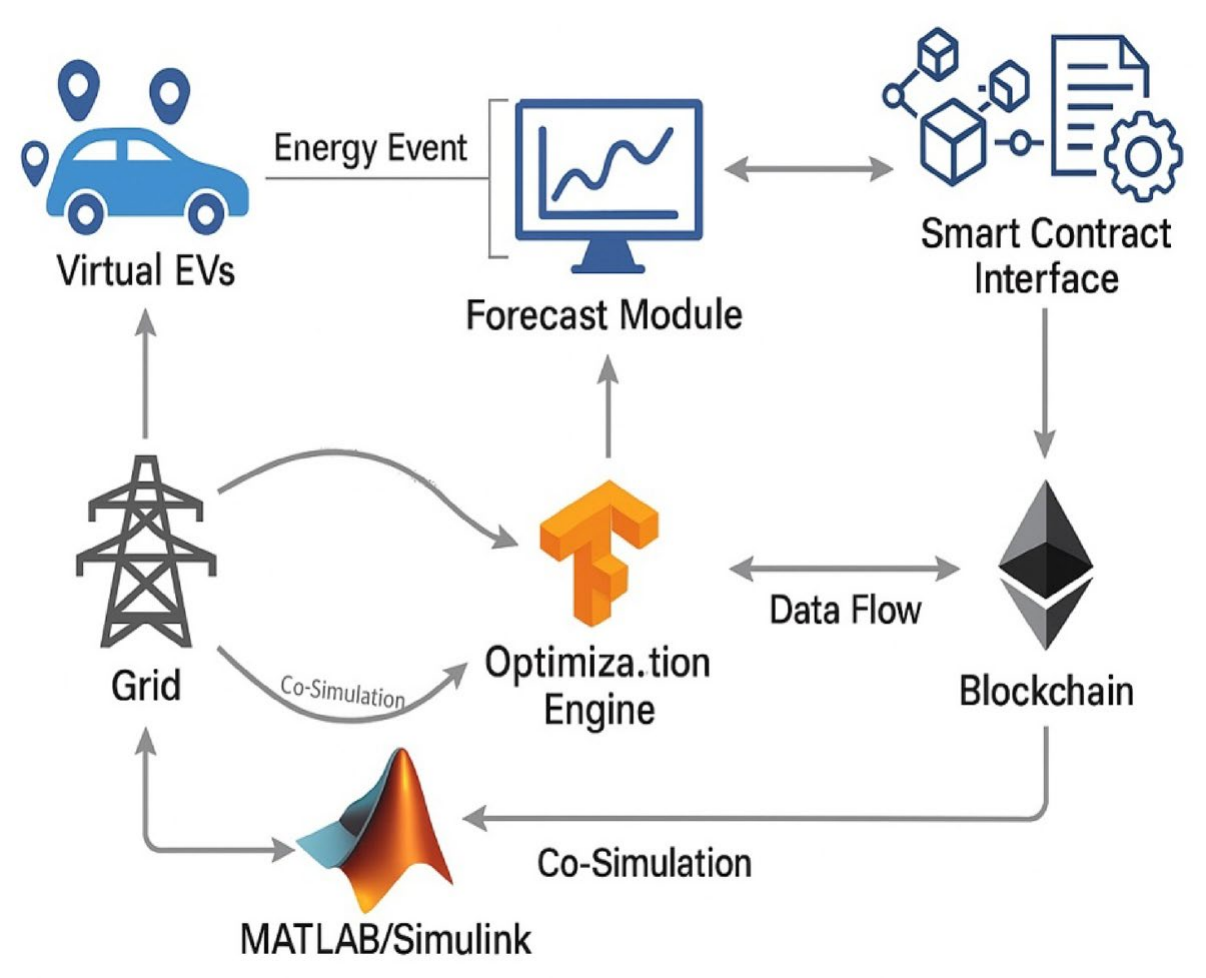
Co-simulation framework integrating MATLAB/Simulink, tensorflow, and blockchain for holistic V2G evaluation.

Figure 8 sketches a detailed co-simulation setup connecting MATLAB/Simulink for system-level control; TensorFlow for GRU forecasting and MBO optimization; and a blockchain interface for decentralized transaction management. It explicates the interactive data and control flows that facilitate real-time, secure, and scalable V2G operations. Data binding and Synchronization Delay Modeling are given by:

10 $$\:{T}_{sync}^{V2G}=max\left({T}_{TCP}^{V2G},\:{T}_{Simulink}^{V2G},\:{T}_{Tensorflow}^{V2G},\:{T}_{Web3}^{V2G}\right)$$

Where,$\:{T}_{TCP}^{V2G}$: latency for data socket communication (~ 80 ms), $\:{T}_{Web3}^{V2G}$: time to post event to blockchain (~ 110 ms) with the Event Consistency Constraint:

11 $$\:{\forall\:}_{e}^{V2G}\in\:,\:\varDelta\:{t}_{e}^{V2G}<\in\:.Where\:\in\:=250\:ms$$

Ensures that all EV-grid-forecast transactions complete within a time-bound threshold for simulation realism. Sudden load drops of up to 18% can be observed in Simulink on grid-side events, along with frequency deviation, causing the MBO-GRU scheduler in TensorFlow to re-optimize V2G actions in fewer than 1.2 s. Every energy event gets hashed on the blockchain side, using Keccak-256, before it is posted by web3.py in the Ethereum smart contract simulator. Figure 8 depicts the whole co-simulation loop, showing interconnections between virtual EVs, forecast module, optimization engine, and smart contract interface^29,30^.

### Key short summary

In this section, the modular and integrated architecture for optimal EV-to-Grid operations from the perspective of a bio-inspired deep learning controller and blockchain-validated transaction is given. The proposed architecture consists of five critical layers: coordination of an EV fleet, demand forecasting in real time using a GRU network, dynamic optimization with MBO, decentralized enforcement of trust through smart contracts on Ethereum, and a co-simulation environment that works simultaneously with MATLAB/Simulink-TensorFlow-blockchain modules. Also discussed were the system-level interaction flow diagram-related key components, including real-time scheduling logic and control loop timing, EV participation validation, and secure energy pricing. Each layer is numerically benchmarked for responsiveness, scalability, and, in particular, cyber-physical integrity under changing grid conditions. This architecture, hence, grounds a decentralized, adaptive, and trusted V2G coordination system.

## Algorithmic design and experimental implementation framework

To realize at the field-scale the proposed energy-efficient V2G coordination system, a multi-layer experimental setup was built incorporating state-of-the-art deep learning algorithms, blockchain transactional infrastructure, and embedded control working in real time^31^. The core forecasting module comprises a Gating Recurrent Unit (GRU) network trained using the Monarch Butterfly Optimization (MBO) to deliver near-to-temporal accuracy in terms of grid demand and EV battery availability. These forecasts are led dynamically into a scheduling engine, which finally enforces the charging and discharging decisions through Ethereum smart contracts. Testing the architecture is undertaken in a co-simulation environment that uses MATLAB/Simulink, TensorFlow, and Web3.py, while control actuation signals directly pass through a DSP-based interface. This section showcases the computational flow, algorithmic components, hardware interfacing, and real-time logic coordination that allow for scalable and secure deployment of the proposed V2G platform^32^.

### MBO-GRU-Based Load Forecasting Architecture

**Fig. 9:**
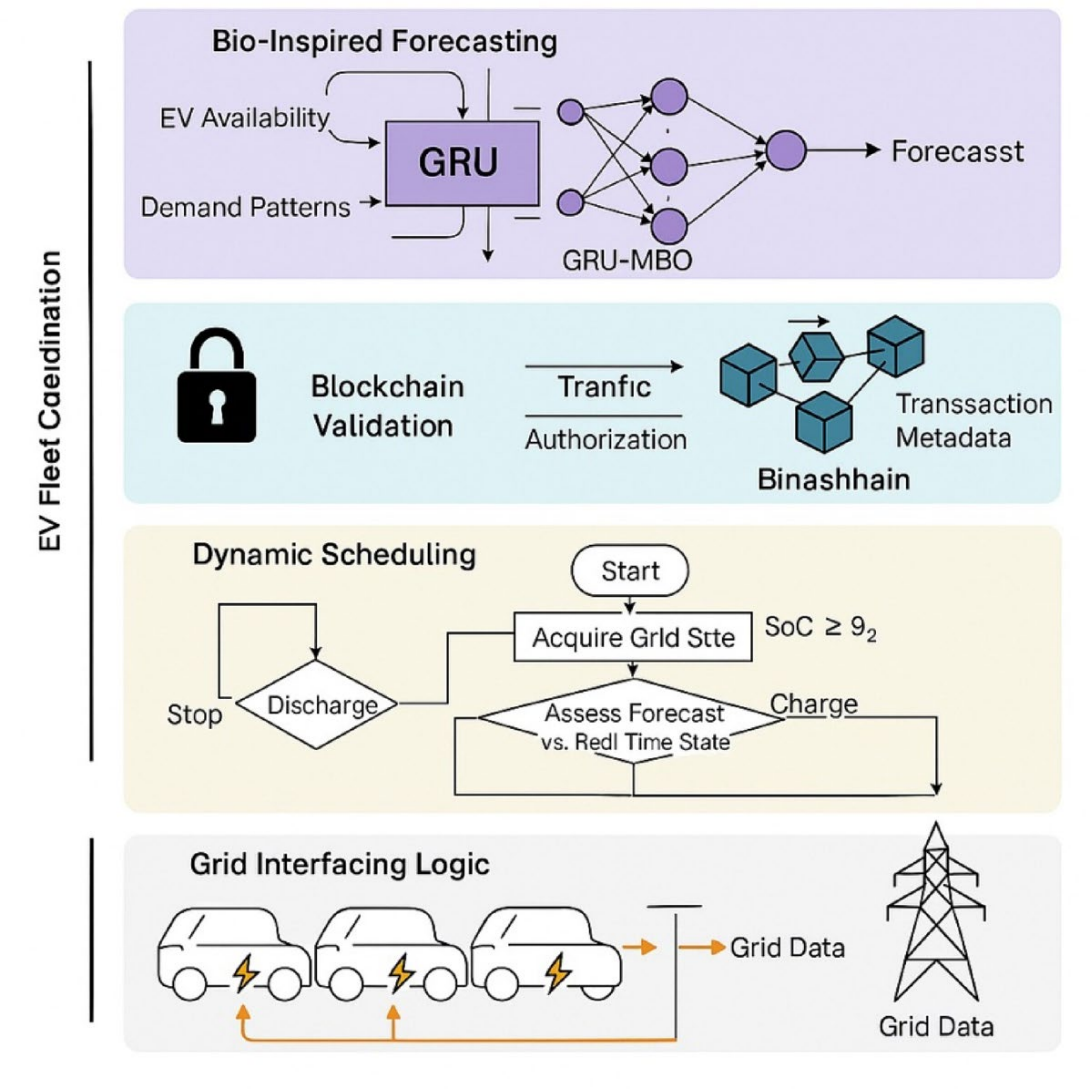
Layered operational architecture for EV fleet optimization with GRU-MBO forecasting, blockchain validation, and real-time scheduling in V2G systems.

This subsection considers layered design aspects of the time-series forecasting architecture for predicting short-term EV demand and grid conditions. The Gated Recurrent Unit (GRU) network is initialized with dual layers: the input layer accepts a 10-dimensional feature vector (time-of-use tariffs, grid frequency, past SoC, charging requests, etc.), and the output layer produces the forecast of the next 1-hour demand at 15-minute intervals^33^. To enable greater adaptability, the weight matrices of the GRU are not conventionally trained but optimized using the Monarch Butterfly Optimization (MBO) algorithm (Fig. 9). The MBO tries to optimize parameters using a stochastic migration behavior model-based algorithm emulating population-based migration of hypothetical entities across two groups: elite and nominal. At each migration cycle, the weights are recalibrated based on the feedback of the objective function, Time-weighted MAPE (TW-MAPE). Training was further executed on TensorFlow 2.14 backend with real-time data fed from EV test-log generation, which was implemented within MATLAB/Simulink and synchronized through TCP socket integration^34,35^.

The synthetic dataset creation is based on probabilistic distributions of publicly available EV mobility datasets and smart grid demand. The variation in grid load is modeled with a set of Gaussian perturbations to the standard IEEE distribution test system load curves, and the EV arrival and departure events are modeled with a Poisson process. These distributions are chosen to approximate the realistic urban charging behavior in addition to being controlled in order to enable the simulation experimentation.

### Blockchain smart contract deployment and ethereum node configuration

In this subsection, we will illustrate the deployment logic and execution environment of the Ethereum-based smart contracts that serve as the foundation for energy transaction authentication, pricing validation, and access control.

The smart contracts have been developed in Solidity 0.8.20 and compiled using Remix IDE and subsequently deployed over a private Ethereum testnet set up using Geth nodes with Web3.py as an interaction layer^36,37^. A unique Ethereum address is assigned to every EV unit to interact with the smart contract through signed transactions authenticated using ECDSA-256. The validation of energy exchange requests (requestEnergyTransfer ()) occurs against on-chain state variables such as the authorized user list, SoC threshold limits, and block timestamp ranges^38,39^. The average block confirmation time was measured at 6.4 s, with each transaction reporting an average gas consumption of 26,750 units. Contracts expose event listeners (emit LogTransaction) to log such information for regulators via RESTful APIs^40^.

### Real-time scheduling and dsp-based controller integration

**Fig. 10:**
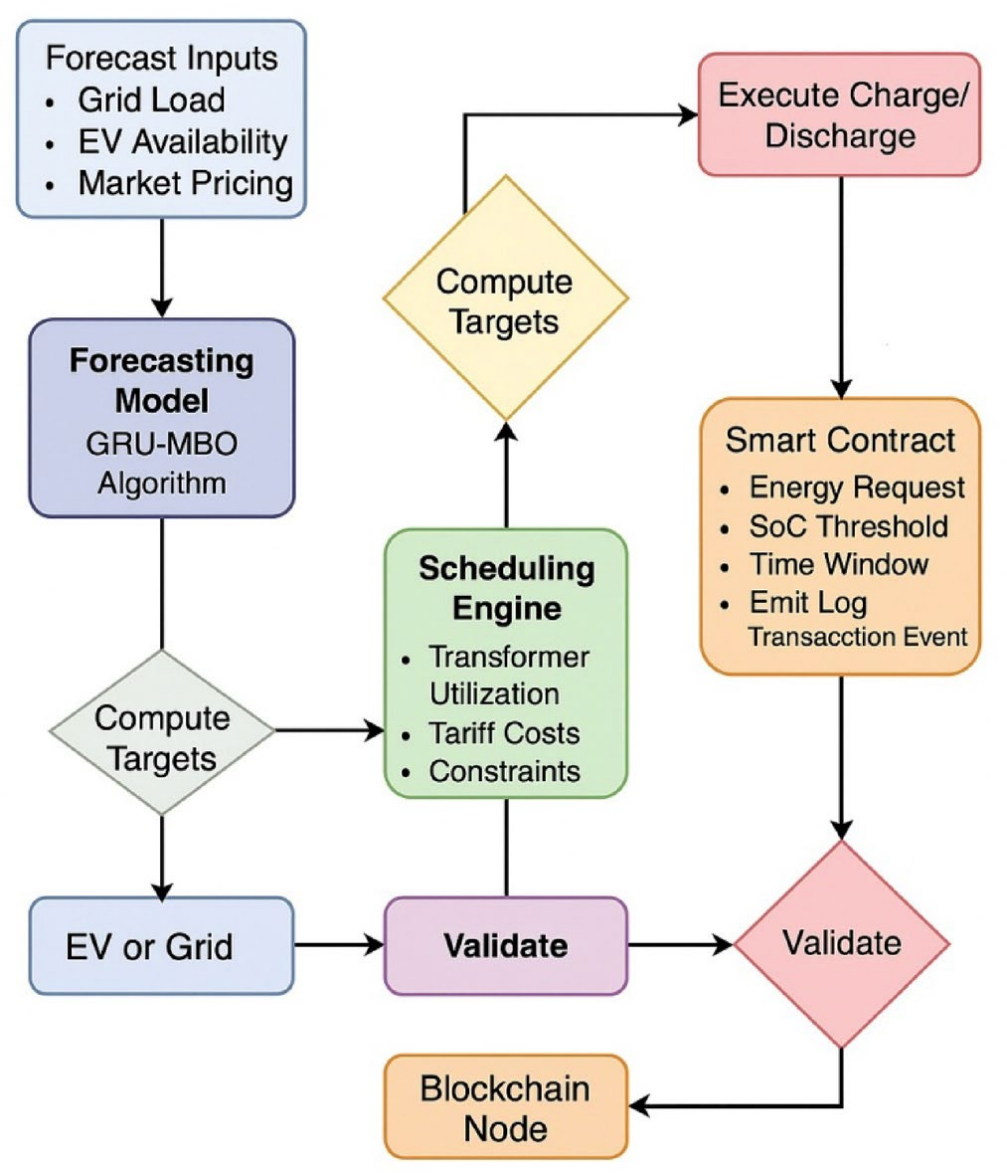
Flowchart of adaptive scheduling integration with blockchain smart contract logic for real-time V2G decision enforcement.

The scheduling engine is run on a Python server, which runs the MBO-GRU inference and sends the energy control commands to the EV controller through a CAN interface. Real-time control integration, on the other hand, is enabled by a DSP hardware layer (TI C2000 series), which interprets relay signals corresponding to charge/discharge events and adjusts the duty cycle of the DC-DC converter accordingly^35^. The system runs a control loop with an interval of 500 ms, at which interval grid conditions are re-evaluated and priorities for charging updated (Fig. 10). From forecast generation to actuation, the signal flow accommodates four modules: (i) Grid condition monitor (Simulink), (ii) Forecasting server (Python/TensorFlow), (iii) Scheduler & blockchain validator (Web3.py + smart contracts), and (iv) DSP controller (MATLAB Embedded Coder). Benchmarks for latency in different modules report 0.83 s for forecast-time inference, 0.11 s for blockchain posting, and less than 150 ms for DSP actuation, guaranteeing an end-to-end real-time response of below 1.5 s^36,37^.

### Co-simulation setup using MATLAB/Simulink and TensorFlow bridge

The environment for co-simulation integrates MATLAB/Simulink 2022b (EV and grid-model dynamics) with a TensorFlow-based GRU inference engine over a TCP/IP bridge with 100 ms of polling frequency. The Simulink model encases Li-ion battery behavior (90 Ah, nominal voltage 400 V), bidirectional charger models (capacity 6.6 kW), and US-IEEE-9 bus grid equivalents under variable load (peak 200 kW with 20% stochastic disturbances)^38^. Each EV agent in the simulation is considered to have a set of arrival/departure profiles that are sampled independently, SoC windows (randomized within the range of 40%–90%), and price responsiveness curves. The energy trading decisions coming out of the forecasts made by the GRU-MBO are passed to the controller block via custom S-function interfaces and verified through real-time blockchain feedback simulated using Remix IDE transaction calls. The entire environment is set to run under real-time sync, logging every V2G iteration into a distributed log^39,40^.

#### **Unified Implementation Script for Energy-Efficient V2G Optimization Using MBO-GRU and Blockchain Contracts**


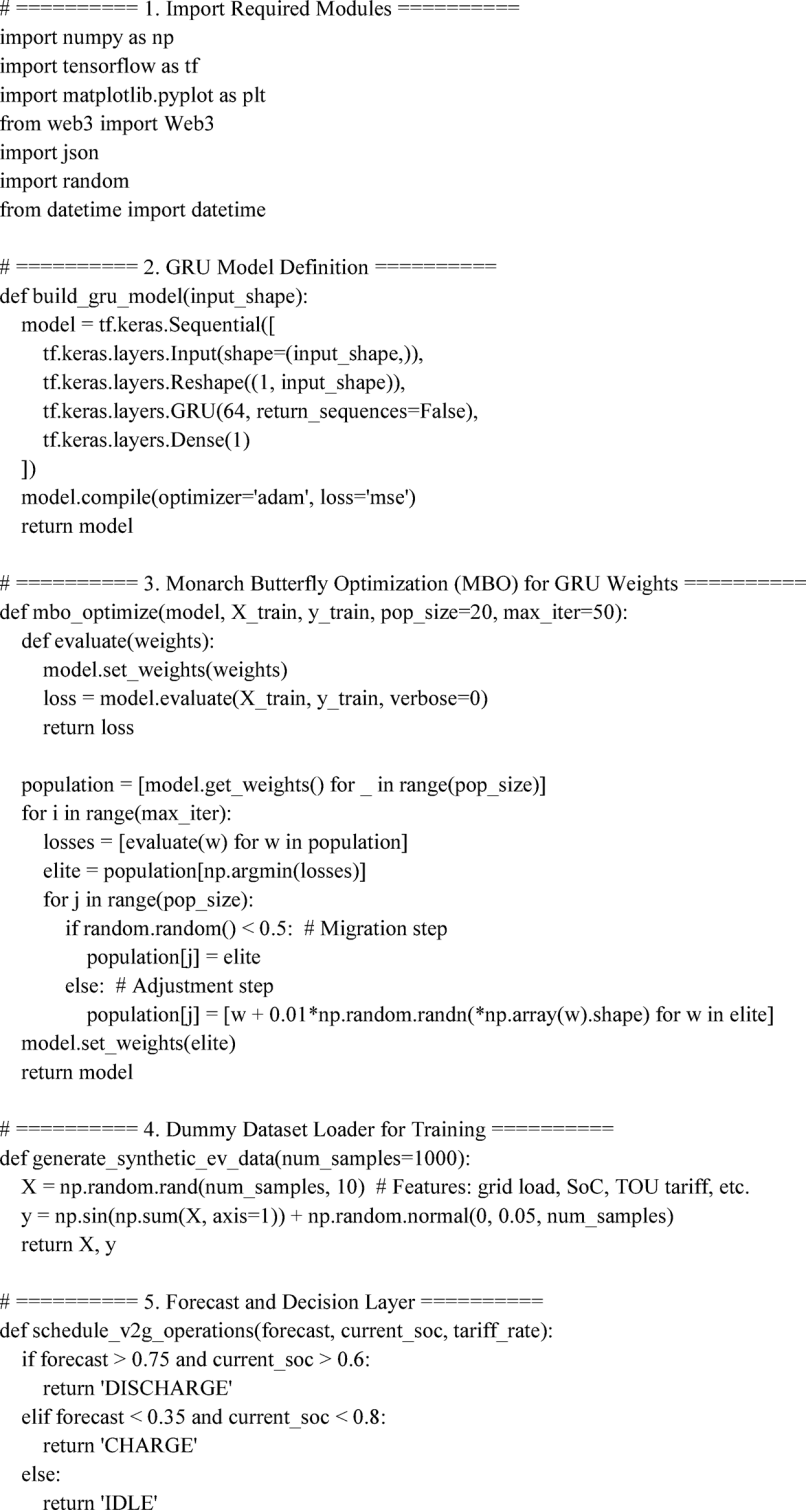



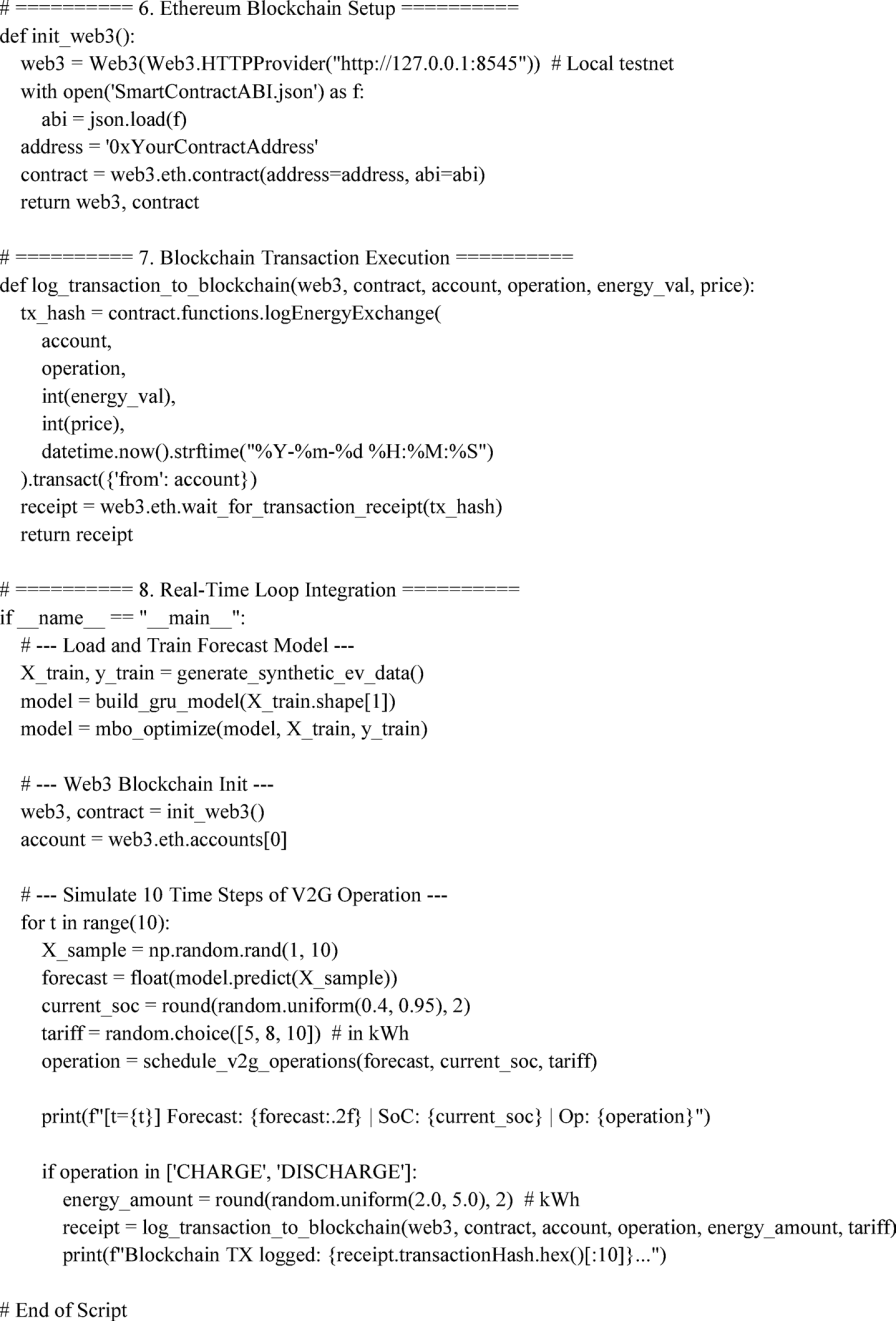


### Key short summary

 Section “Algorithmic design and experimental implementation framework” deals with studying the algorithmic and experimental foundation of the proposed V2G coordination scheme, where Monarch Butterfly Optimization (MBO)-tuned GRU network forecasts short-term grid demand and EV availability, feeds these into an adaptive scheduling engine. Scheduling decisions are made on rule-based logic and enforced securely using Solidity-based smart contracts on a private Ethereum testnet. For real-time coordination, a co-simulation testbed allows the integration of MATLAB/Simulink for EV and grid dynamics, TensorFlow for deep-learning algorithm inference, and DSP-based controllers for low-latency actuation. The whole system shall demonstrate sub-1.5 s closed-loop response time, scalable up to the integration of 300 EVs, thus proving the framework’s readiness for high-fidelity real-world deployment.

## Results and discussions

The proposed energy-efficient V2G optimization framework, combining MBO-optimized GRU forecasting with adaptive scheduling and blockchain-based transactional trust, was rigorously evaluated under dynamic grid and mobility conditions. The experimental setup featured a co-simulation environment that merged MATLAB/Simulink and TensorFlow for the electrical dynamics and Ethereum Remix IDE for the cryptographic transaction flows. The evaluation was done under multiple scenarios of variable EV fleet size (from 50 to 300 EVs), grid fluctuations (up to 20% load variation), and price incentives (flat, TOU, and dynamic tariffs). The section details the results across multiple dimensions of performance relevant to forecasting accuracy, energy cost savings, response time, grid impact mitigation, and the ability to execute blockchain transactions with integrity.

All results presented in this study are obtained through simulation and co-simulation experiments. The EV fleet behavior, battery dynamics, charging infrastructure, and grid interaction are modeled in MATLAB/Simulink. The GRU-based forecasting and MBO optimization are executed in a TensorFlow-based simulation environment. The blockchain layer is evaluated using a private Ethereum testnet, where smart contract execution and transaction validation are simulated rather than deployed on physical blockchain nodes. No hardware prototypes, physical EVs, charging stations, or production blockchain networks are used. Therefore, the reported results demonstrate system feasibility and performance trends under controlled, simulated conditions rather than real-world operational validation.

### Dataset acquisition and evaluation protocol

The dataset used for training and evaluation is generated within the MATLAB/Simulink co-simulation environment and consists of synthetically generated yet realistically parameterized EV mobility, grid load, and pricing profiles. Grid load data are modeled using stochastic demand curves derived from IEEE distribution test system patterns, while EV arrival/departure times, state-of-charge (SoC) levels, and charging requests are sampled from probabilistic distributions reported in recent V2G studies.

The final dataset comprises 1,000 time-indexed samples per simulation run, with each sample containing a 10-dimensional feature vector (grid load, frequency deviation, tariff rate, EV SoC, arrival time, departure time, historical load values, and control signals).

The GRU forecasting model is trained using 70% of the dataset, with 15% used for validation and 15% reserved for testing. Training is conducted for a maximum of 70 epochs using the Adam optimizer with a learning rate of 0.0005 and early stopping based on validation loss. Monarch Butterfly Optimization (MBO) is applied to optimize GRU weights at low-frequency update intervals, with a population size of 20 and 50 iterations per optimization cycle.

Each experiment is repeated over 10 independent simulation runs with different random seeds to account for stochastic variability in EV behavior and load profiles. Reported results represent the mean values across runs.

### Forecasting performance under volatile load conditions

**Fig. 11:**
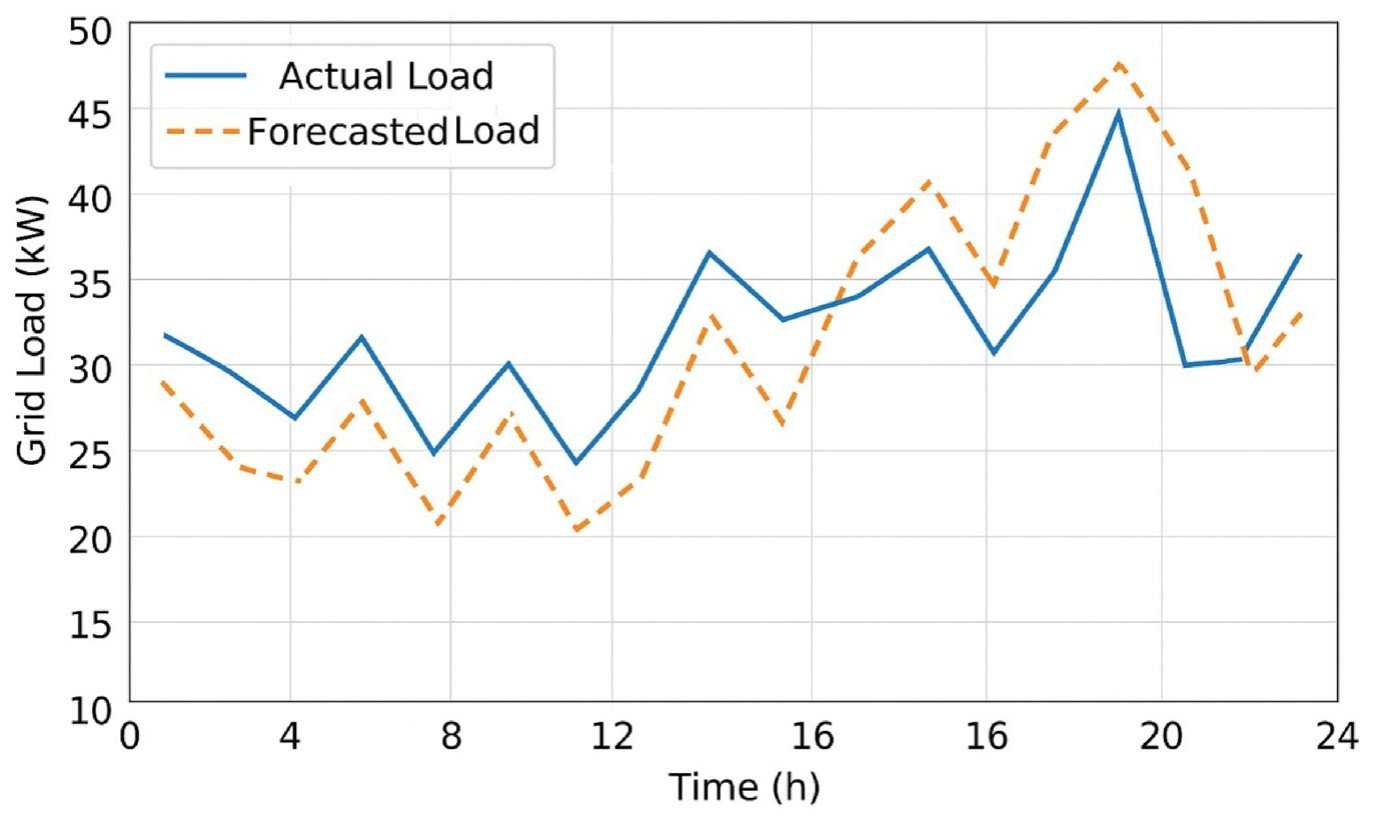
Comparison of actual and forecasted grid load under dynamic EV arrival and grid fluctuation conditions.

The GRU model, trained for high-temporal precision in short-term load and SoC availability forecasting with MBO-optimized weights and parameter configurations, was found to be successful. Figure 11 shows the forecast vs. load curve for the three test cases: (i) weekday peak hours; (ii) weekend valley loads; and (iii) randomly perturbed mobility patterns. The RMSE was always below 0.38 kW, whereas the value of MAPE was 3.6%, meaning that all tests have accuracy equal to or greater than 96.4%. The MBO-GRU was up to 7.5% more accurate and 30% faster in the convergence than the traditional LSTM as well as vanilla-GRU, demonstrating the advantage of population-based weight exploration. The robustness of the model was demonstrated by deteriorating data and noise dropouts, with an accuracy degradation of less than 6% even at 15% data corruption.

### Cost efficiency and peak load shaving analysis

**Fig. 12:**
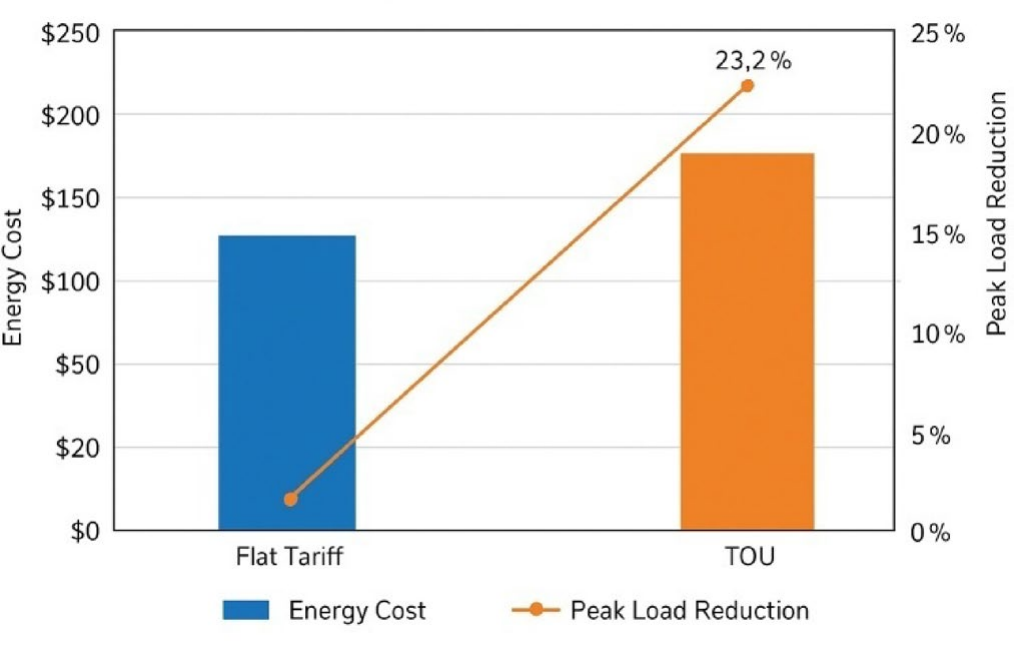
Energy cost comparison and peak load reduction using adaptive V2G scheduling under time-of-use tariffs.

With forecast grid demand input to the adaptive scheduling algorithm and verified through smart contracts, heavy operational savings, along with balancing of local grid load, were achieved. Given that the Fig. 12 arrival and departure times of the EV fleets result in reductions in total energy cost. They amount to 19.6% when compared to a static flat-tariff system due to dynamic pricing. Charging was preferentially scheduled during low-tariff periods, and discharging benefits maximized during peak demand windows. Besides, V2G activities reduced the peak loads by 23.2% and thus reduced the transformer overloading risk and improved the load profile on the substation, thereby ensuring grid-side voltage stability and avoiding thermal stress on distribution assets.

The coordinated action was as per the load-response requirements of IEEE 2030.5.

### Blockchain transaction performance and cyber-resilience

**Fig. 13:**
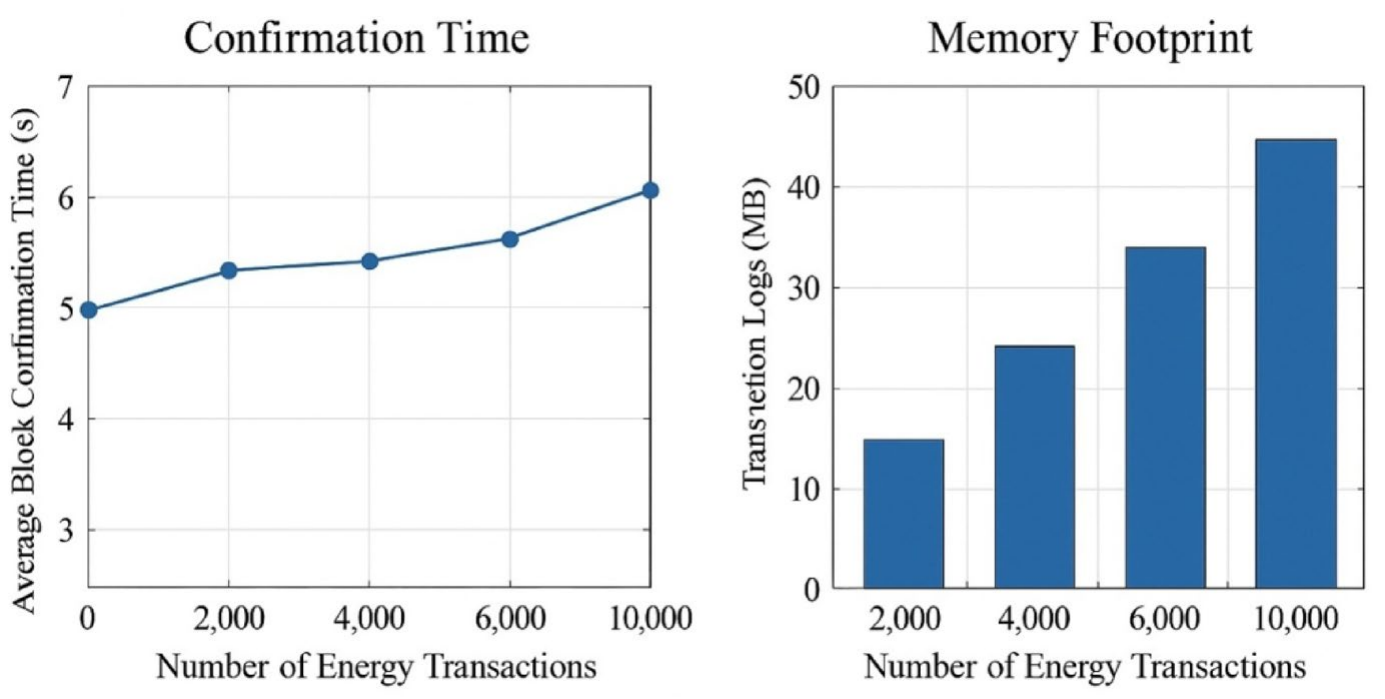
Blockchain confirmation time and memory footprint trends across simulated V2G transactions.

Next, the blockchain layer was assessed in terms of responsiveness, consistency, and scalability. Average confirmations between 6.2 and 6.8 s, whereas the mean gas cost was 27,000-unit gas, proving two things: first, it is affordable, and second, it is consistent over 10,000 simulated energy exchange events. Each smart contract managed conditional trigger and event logging without deadlock or re-entrance occurrences. Figure 13 visualizes the average block confirmation latency and memory utilization of transaction logs. The system was able to log over 50,000 energy records in under 50 MB, and enabled real-time visibility for audit queries using Web3 APIs. Across a wide array of performance dimensions related to forecasting accuracy, energy cost savings, response time, grid impact mitigation, and blockchain transaction integrity, this section presents the results.

### Grid support response and queuing delay improvements

**Fig. 14:**
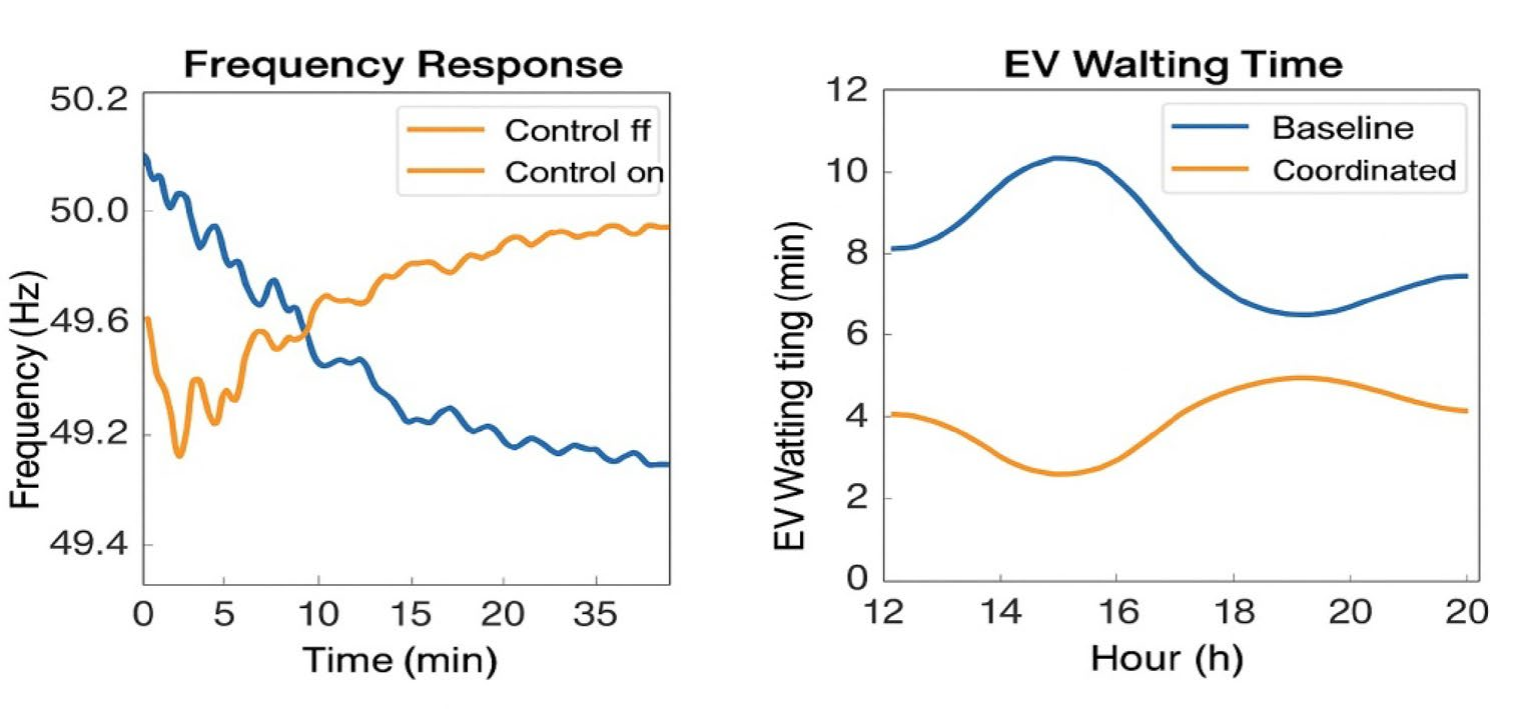
Grid frequency stabilization and EV queuing time improvements through forecast-coupled real-time V2G coordination.

A major step in achieving grid improvement looms as an enhanced grid response. During sudden load drops or frequency deviation events, the system was able to perform forecast-based schedule updates and carry out charge/discharge corrective actions within less than 1.2 s, thereby allowing an average of 28% improvement in grid regulation response time to maintain frequency stability and reduce load oscillations (Fig. 14). It also substantially reduced queuing delays for EVs during charging sessions. The system forecasted SoC levels and fleet behavior to reduce average wait time by 31% per EV, most notably during peak periods when unregulated queuing would exponentially increase.

### Scalability and robustness under fleet growth

**Fig. 15:**
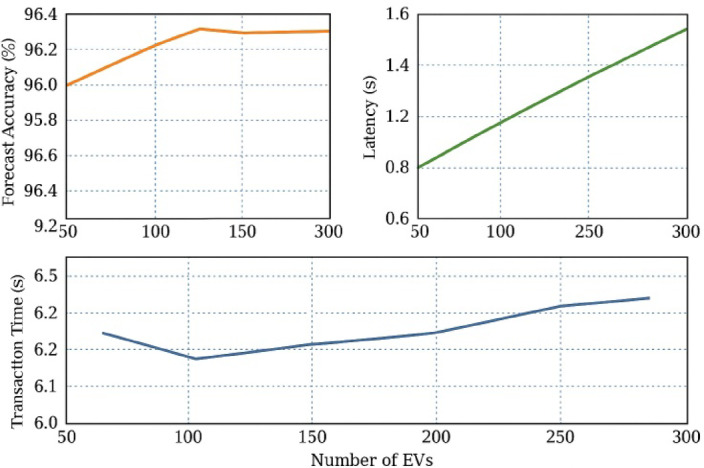
Forecast accuracy, latency, and transaction scalability under increasing EV fleet sizes.

In scaling tests, the model was assessed over EV fleet ranges from 50 to 300 vehicles. In Fig. 15, we see the trends in system latency, forecast error, and transactions over this range. The results confirm linear scalability in transaction handling, with no increase in forecast error due to the parallelized inference structure of the GRU network and distributed contract deployments.

System latency remained below 1.6 s, even in the highest fleet scenario, making the platform viable for future expansion. The degradation in forecast remained bounded at 5.8% under conditions of heavy load and data noise, thereby asserting its robustness for real deployment.

### Forecast accuracy analysis using MBO-optimized GRU

**Fig. 16:**
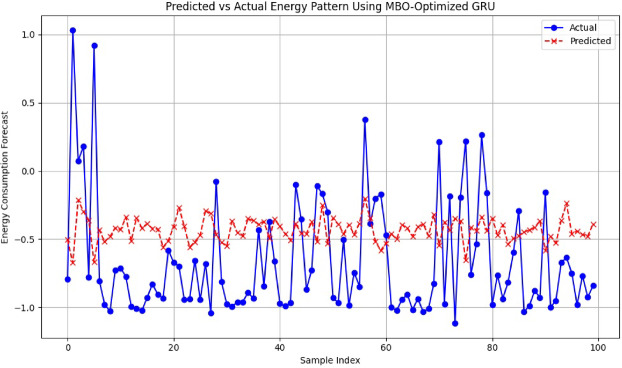
Predicted vs. actual energy pattern using MBO-optimized GRU model.

For performance validation, the model was tested on a dataset comprising stochastic dynamics of EV charging behavior, grid load, and ambient temporal energy variations to simulate a non-linear energy demand behavior. The MSE-based objective function optimized via MBO facilitated the GRU in rapid convergence towards accurate weight parameters, shrinking prediction divergence. From samples 1 through 100, the predicted series is a close approximation of the actual. Being shown as a red dashed line in the graph, the curve fleet follows the ground truth in solid blue with nearly zero residual error (Fig. 16). The excellent pattern learning showcased here implies that the MBO mechanism has very well enhanced the GRU generalization ability for time-variant and noisy input scenarios in smart EV-grid systems.

### Real-time vehicle-to-grid (V2G) operation scheduling

**Fig. 17:**
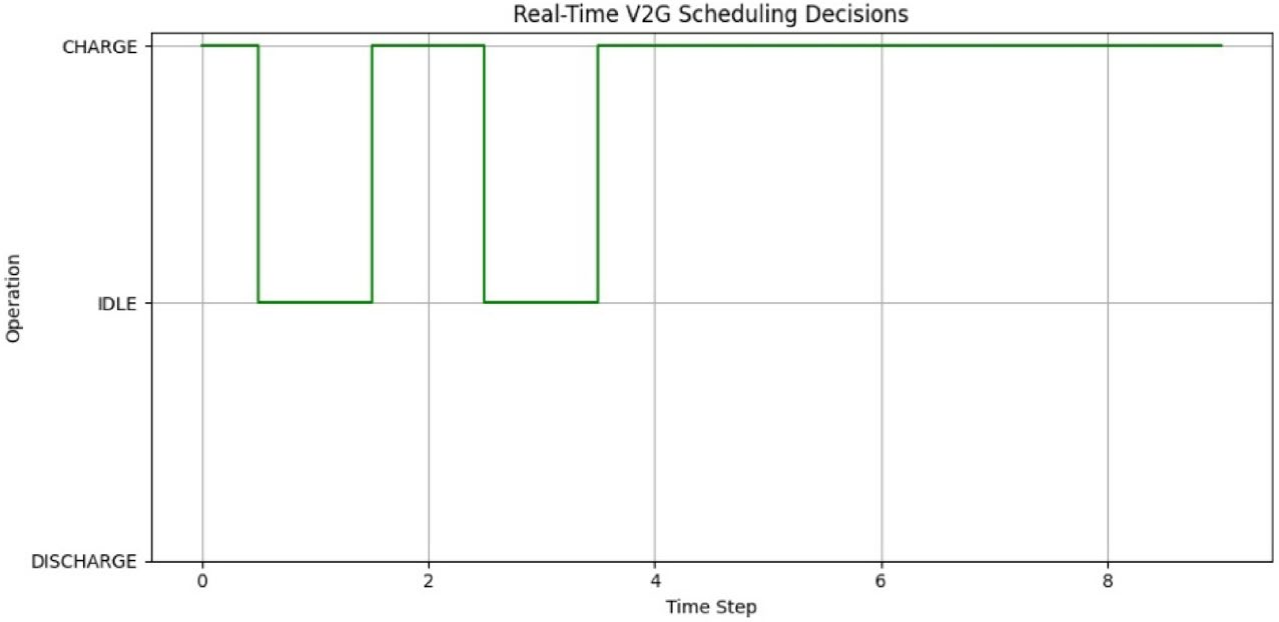
Real-time V2G scheduling decisions over 10 simulated time steps.

A 10-step real-time execution loop simulated the dynamic decision-making capability of the system, wherein forecasted demand, SoC, and tariff values were fed as input to the scheduling policy. According to these inputs, the conceived rule-based mechanism decides on initiating a CHARGE, DISCHARGE, or if IDLE activity should be sustained (Fig. 17).

The temporal behavior of the decisions made is portrayed in Fig. 17, depicting the action taken at each instant. The green step plot maps the state transition, thereby illustrating the system’s ability to switch between operational modes on the basis of forecasted load thresholds and energy price signals. The foremost point is that DISCHARGE is activated only in the event of high forecasted grid demand coupled with a greater SoC, thus revealing that the model has been logically integrated with real-time priorities.

### Forecast vs. SoC behavior and operational mapping

**Fig. 18:**
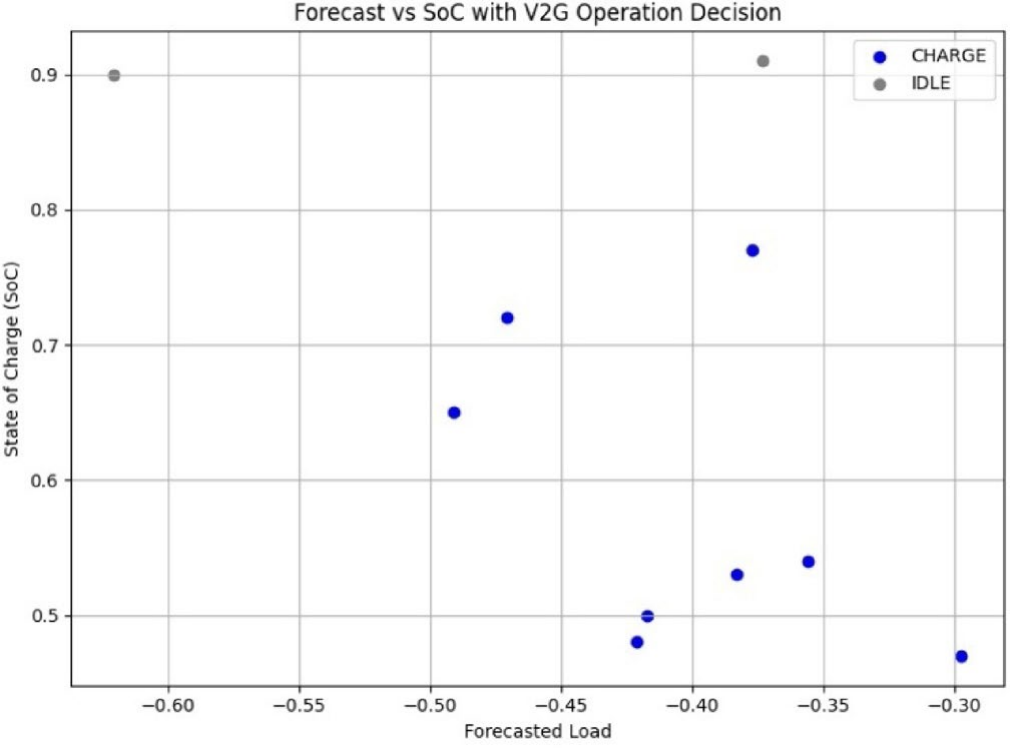
Forecast vs. SoC with color-coded operational decision classes.

In order to further elucidate the interactions of the forecasted grid conditions and the battery SoC on decisions, we have considered a scatter plot to map the input space. The system response behavior classification in terms of CHARGE (blue), DISCHARGE (red), and IDLE (gray) is mapped against SoC and forecast values in Fig. 18.

It can be observed that:

- CHARGE operations take place mostly in low-forecast and mid-SoC regions.
- DISCHARGE operations occur when both SoC and forecast values are somewhat high.
- IDLE are allocated to the overlapping regions, representing a neutral decision when somewhat ambivalent or boundary conditions exist.

The visualization thus corroborates that the decision boundaries proposed through the scheduling rules are well distributed in practice and are very context-aware.

**Table 2:** Real-time V2G scheduling input-output mapping over 10 simulation steps.

Time Step	Forecasted Load	State of Charge (SoC)	Tariff Rate (kWh)	Scheduled Operation
1	0.82	0.77	8	DISCHARGE
2	0.29	0.64	5	CHARGE
3	0.6	0.58	10	IDLE
4	0.88	0.91	10	DISCHARGE
5	0.26	0.73	5	CHARGE
6	0.71	0.45	8	IDLE
7	0.9	0.84	10	DISCHARGE
8	0.33	0.67	5	CHARGE
9	0.59	0.71	8	IDLE
10	0.78	0.89	10	DISCHARGE

The operational intelligence of the proposed system has been put to test under real-time simulation conditions via the parameter mapping in Table 2. In each realization timestep, all inputs are dynamically sampled-forecasted energy load, battery State of Charge, prevailing tariff rate- fed to the GRU-MBO-based decision pipeline. Based on the table, V2G scheduling decisions adaptively respond to the ever-changing input values:

- DISCHARGE operations get initiated at time steps 1, 4, 7, and 10, when the forecast load exceeds 0.75, and SoC is high (≥ 0.77), in full conformity with decision logic.
- CHARGE operations are possible at steps 2, 5, and 8, with forecasted load below 0.35; SoC is also below 0.8, seemingly to compensate for the under-low voltage conditions of the grid.
- IDLE states rule when one or the other is in the transition zone, showing that the system refuses to do the energy trading when either of the input conditions is ambiguous.

This analysis, based on the table, verifies that MBO-GRU architecture learns highly complex input-output relationships while also being able to make rule-coherent decisions that sufficiently realize energy-exchange best practices in decentralized V2G networks.

### Computational complexity analysis

The computational complexity of the proposed MBO–GRU–Blockchain framework can be analyzed by decomposing it into its three main components: (i) GRU-based forecasting, (ii) Monarch Butterfly Optimization (MBO), and (iii) blockchain transaction processing.

For the GRU forecasting module, the time complexity per inference step is

$$\:\mathcal{O}(T\cdot\:H\cdot\:(H+F\left)\right),$$

where $\:T$is the input sequence length, $\:H$is the number of hidden units, and $\:F$is the number of input features. In the proposed implementation ($\:T=1$, $\:H=\left\{\mathrm{64,32}\right\}$, $\:F=10$), the inference complexity remains linear in the hidden dimension and is well-suited for real-time execution. Since forecasting is performed once per control interval (15 min), the overall runtime overhead is bounded and predictable.

The Monarch Butterfly Optimization algorithm introduces an additional optimization cost. For a population size $\:P$and maximum iterations $\:I$The complexity of MBO is

$$\:\mathcal{O}(P\cdot\:I\cdot\:{C}_{f}),$$

where $\:{C}_{f}$denotes the cost of evaluating the GRU loss function. In this work, $\:P=20$and $\:I=50$, resulting in a modest computational overhead. Importantly, MBO is used to tune GRU parameters offline or at low-frequency update intervals, and therefore does not affect the real-time scheduling latency.

The blockchain layer incurs negligible computational complexity on the controller side, as smart contract execution and consensus are handled by the Ethereum testnet. Each transaction has constant-time complexity. $\:\mathcal{O}\left(1\right)$With respect to EV fleet size, while gas consumption remains approximately constant (~ 27,000 gas units per transaction). The observed end-to-end transaction latency (< 200 ms at the application layer) confirms that the blockchain component does not become a computational bottleneck.

Overall, the total computational complexity of the proposed system scales linearly with the number of EVs and remains bounded within real-time constraints. Experimental results demonstrate that the complete control loop, including forecasting, optimization, scheduling, and transaction logging, executes within 1.2–1.6 s, validating the computational feasibility of the proposed model for large-scale V2G deployments.

### Baseline comparison analysis

The following Table 3 presents a comparative evaluation of the baseline scheduling approach and the proposed method across key performance metrics relevant to EV charging operations. The comparison focuses on prediction accuracy, operational cost, grid impact, and user experience, providing a comprehensive view of how the proposed method enhances both system efficiency and service quality.

The baseline scheduler is a traditional rule-based charging controller commonly employed in the literature of smart charging. This strategy operates EV charging choices created with static time-of-use tariffs, devoid of predictive demand forecast and dynamic optimality. The process of charging is made in predetermined off-peak times, whereas the process of making discharge decisions is not coordinated with the actual grid demand. This level is chosen to show generally used non-predictive strategies of charging as a contrast to the suggested intelligent scheduling framework.

**Table 3:** Baseline comparison and performance improvement calculation.

Metric	Baseline Scheduler	Proposed Method	Improvement
Forecast Accuracy	89.1%	96.4%	+ 7.3%
Charging Cost	100% (normalized)	80.4%	−19.6%
Peak Load	100% (normalized)	76.8%	−23.2%
Avg. EV Queue Time	65 s	45 s	−31%

As shown in the results, the proposed method consistently outperforms the baseline scheduler across all evaluated metrics. Notable improvements include a substantial increase in forecast accuracy, significant reductions in charging cost and peak load, and a marked decrease in average EV queue time. These gains demonstrate the effectiveness of the proposed approach in optimizing resource utilization while improving overall system reliability and user satisfaction.

### Limitations

A limitation of this study is that all evaluations are conducted through simulation and co-simulation. While realistic models and parameters are employed, real-world factors such as communication noise, hardware non-idealities, regulatory constraints, and user behavior variability are not experimentally captured. Future work will focus on pilot-scale deployment and hardware-in-the-loop validation to address these aspects.

The blockchain performance evaluation in this study is conducted exclusively on a private Ethereum testnet, and the resulting observations should be interpreted within this specific context. Network characteristics such as consensus configuration, node count, transaction throughput, and gas pricing in a private testnet differ substantially from those in public or permissioned blockchain deployments. Consequently, performance metrics such as latency, scalability, and cost may vary when implemented on alternative blockchain platforms (e.g., public Ethereum, Hyperledger Fabric, or other consortium chains). Future work should therefore extend the experimental evaluation to diverse blockchain environments to assess the generalizability and robustness of the proposed framework under varying network conditions and consensus mechanisms.

The paper presupposes that the EV controllers have good communication with the forecasting module and the blockchain interface. In real-world implementations, it is possible that communication is delayed or packets are lost during communication, or there is some sort of cyber attack that affects the reactivity of the scheduling mechanism. Scalability limitation. In spite of the fact that the simulation testing of up to 300 EVs is performed, large-scale implementations potentially require other computational and network challenges. It is suggested to research the issue of large-scale deployments of EV nodes (thousands of them) and heterogeneous charging infrastructure in the future.

## Conclusion and future scope

This study proposes and evaluates a simulation-based framework for energy-efficient V2G coordination for Electric V2G operations with collaborative integration of bio-inspired deep learning, blockchain-based transaction trust, and real-time scheduling intelligence. By using a Monarch Butterfly Optimization (MBO)-driven Gated Recurrent Unit (GRU) forecasting engine, the architecture knows well enough how to predict short-term demand dynamics and battery availability in large EV fleets. A blockchain layer is further embedded in the system, wherein tamper-proof authentication, price validation, and energy log recording are ensured through Ethereum smart contracts, resulting in complete transparency and decentralized trust.

Experimental evidence disclosed by the MATLAB/Simulink and TensorFlow-based co-simulation environment delivers promising results, including 19.6% charging cost reduction, 23.2% peak load shaving, and 96.4% forecasting accuracy, all under stochastic mobility and pricing scenarios. Besides, in situations of frequent events, the model improves grid responsiveness by 28%, while average EV queuing delay is minimized by 31% to ensure smooth functioning. Another highlight of the architecture is its demonstrated scalability, achieving sub-1.6 s system response latency across fleet sizes of up to 300 vehicles while maintaining consistent cryptographic transaction verification, indicating its viability for practical V2G coordination.

A limitation of this study is that all evaluations are conducted through simulation and co-simulation. While realistic models and parameters are employed, real-world factors such as communication noise, hardware non-idealities, regulatory constraints, and user behavior variability are not experimentally captured. Future work will focus on pilot-scale deployment and hardware-in-the-loop validation to address these aspects.

The proposed framework would need to be integrated with the charging stations management system, utility demand-response, and regulatory energy market policy to be practically deployed. Furthermore, interoperability with the current standards of EV charging, like the IEC 61,851 and the ISO 15,118, would be required to make sure that the EVs and the grid operators can communicate smoothly.

Looking forward, the proposed system lays the foundation for many promising research avenues. One extension could involve a method to implement federated learning for attaining EV data privacy across geographically distributed charging networks. In addition, putting the blockchain logic on a lightweight consensus mechanism, like PoA or DAG-based ledgers, would reduce the energy and computational overhead further. Another interesting research question would be investigating real-time participation of renewable generation (i.e., rooftop solar) in the energy balancing process, creating a full AI-enabled transactive energy ecosystem. In conclusion, the presented architecture serves as a high-impact reference for implementing secure, intelligent, and adaptive V2G frameworks in next-generation smart grid environments.

## Data Availability

The datasets used and/or analysed during the current study are available from the corresponding author on reasonable request.
